# Sparse Multicategory Generalized Distance Weighted Discrimination in Ultra-High Dimensions

**DOI:** 10.3390/e22111257

**Published:** 2020-11-05

**Authors:** Tong Su, Yafei Wang, Yi Liu, William G. Branton, Eugene Asahchop, Christopher Power, Bei Jiang, Linglong Kong, Niansheng Tang

**Affiliations:** 1 Key Lab of Statistical Modeling and Data Analysis of Yunnan Province, Yunnan University, Kunming 650091, China; sutong_366@sina.com; 2 Department of Mathematical and Statistical Sciences, University of Alberta, Edmonton, AB T6G 2G1, Canada; yafei2@ualberta.ca (Y.W.); yliu16@ualberta.ca (Y.L.); bei1@ualberta.ca (B.J.); 3 Department of Medicine (Neurology), University of Alberta, Edmonton, AB T6G 2G1, Canada; wbranton@ualberta.ca (W.G.B.); asahchop@ualberta.ca (E.A.); cp9@ualberta.ca (C.P.)

**Keywords:** high dimension, multicategory classification, DWD, sparse group lasso, *L_2_*-consistency, proximal algorithm

## Abstract

Distance weighted discrimination (DWD) is an appealing classification method that is capable of overcoming data piling problems in high-dimensional settings. Especially when various sparsity structures are assumed in these settings, variable selection in multicategory classification poses great challenges. In this paper, we propose a multicategory generalized DWD (MgDWD) method that maintains intrinsic variable group structures during selection using a sparse group lasso penalty. Theoretically, we derive minimizer uniqueness for the penalized MgDWD loss function and consistency properties for the proposed classifier. We further develop an efficient algorithm based on the proximal operator to solve the optimization problem. The performance of MgDWD is evaluated using finite sample simulations and miRNA data from an HIV study.

## 1. Introduction

Classification problems appear in diverse practical applications, such as spam e-mail classification, disease diagnosis and drug discovery, among many others (e.g., [1,2,3]). In these classification problems, the goal is to predict class labels based on a given set of variables. Recent research has focused extensively on linear classification: see [4,5] for comprehensive introductions. Among many linear classification methods, support vector machines (SVMs) (see [6,7]) and distance-weighted discrimination (DWD) (see [8,9,10]) are two commonly used large-margin based classification methods.

Owing to the recent advent of new technologies for data acquisition and storage, classification with high dimensional features, i.e., a large number of variables, has become a ubiquitous problem in both theoretical and applied scientific studies. Typically, only a small number of instances are available, a setting we refer to as high-dimensional, low-sample size (HDLSS), as in [11]. In the HDLSS setting, a so-called “data-piling” phenomenon is observed in [8] for SVMs, occurring when projections of many training instances onto a vector normal to the separating hyperplane are nearly identical, suggesting severe overfitting. DWD was originally proposed to overcome data-piling in the HDLSS setting. In binary classification problems, linear SVMs seek a hyperplane maximizing the smallest margin for all data points, while DWD seeks a hyperplane minimizing the sum of inverse margins over all data points. Reference [8] suggests replacing the inverse margins by the *q*-th power of the inverse margins in a generalized DWD method; see [12] for a detailed description. Formally, for a training data set ${\left\{\left(y_{i},X_{i}\right)\right\}}_{i=1}^{N}$ of *N* observations, where $X_{i}\in R^{p}$ and $y_{i}\in \left\{-1,1\right\}$, binary generalized linear DWD seeks a proper separating hyperplane $\left\{X:a+X^{⊤}b=0\right\}$ through the optimization problem

(1) $$\begin{array}{cc} \underset{a,b}{\arg\max} & \sum_{i=1}^{N}\frac{1}{d_{i}^{q}}\\ \;s.t.\;d_{i} & =y_{i}\left(a+X_{i}^{T}b\right)+\eta_{i}\geq 0,\forall i,\\ \eta_{i} & \geq 0,\forall i,\sum_{i}\eta_{i}\leq c,\\ {∥b∥}_{2}^{2} & =1, \end{array}$$

where *a* and b are the intercept and slope parameters, respectively. The slack variable $\eta_{i}$ is introduced to ensure that the corresponding margin $d_{i}$ is non-negative and the constant c>0 is a tuning parameter to control the overlap between classes. Problem (1) can also be written in a loss-plus-penalty form (e.g., [12]) as

(2) $$\begin{array}{c} \left(\hat{a},\hat{b}\right)=\underset{a,b}{\mathrm{argmin}}\left[\frac{1}{N}\sum_{i=1}^{N}\phi_{q}\left\{y_{i}\left(a+X_{i}^{⊤}b\right)\right\}+\lambda {∥b∥}_{2}^{2}\right], \end{array}$$

where

(3) $$\begin{array}{c} \phi_{q}\left(u\right)=\left\{\begin{array}{cc} 1-u, & \;\mathrm{if}\;u\leq Q\\ \varphi_{q}\left(u\right), & \;\mathrm{if}\;u>Q, \end{array}\right. \end{array}$$

with $Q=\frac{q}{q+1}$, q>0 and $\varphi_{q}\left(u\right)=\left(1-Q\right){\left(Qu^{-1}\right)}^{q}$. When q=1, (1) becomes the standard DWD problem in [8] while problem (2) appears in [9,13].

The binary classification problem (1) is well studied. However, in many applications such as image classification [1], cancer diagnosis [2] and speech recognition [3], to name a few, problems with more than two categories are commonplace. To solve these multicategory problems with the DWD classifier, approaches based on either formulation (1) or (2) are common. One common strategy is to extend problem (1) to multiple classes by solving a series of binary problems in a one-versus-one (OVO) or one-versus-rest (OVR) method (e.g., [14]). Instead of reducing the multicategory problem to a binary one, another strategy based on problem (1) considers all classes at once. As shown in [14], this approach generally works better than the OVO and OVR methods. Based on an extension of problem (2), [15] proposes multicategory DWD, written in a loss-plus-penalty form as

(4) $$\begin{array}{c} \min_{a_{k},b_{k}}\frac{1}{N}\sum_{i=1}^{N}\phi_{q}\left(a_{y_{i}}+X_{i}^{⊤}b_{y_{i}}\right)+\lambda \sum_{k=1}^{K}{∥b_{k}∥}_{2}^{2}\\ s.t.\sum_{k=1}^{K}a_{k}=0;\sum_{k=1}^{K}b_{jk}=0,\;\forall j=1,\ldots ,p, \end{array}$$

with $y_{i},\;k\in \left\{1,\ldots ,K\right\}$ and where $a_{k}$ and $b_{k}=\left(b_{1k},\cdots ,b_{pk}\right)$ are the intercept and slope parameters for each category *k*, respectively. Although these methods can be applied to multicategory classification in the HDLSS setting, both problems (2) and (4) use the $L_{2}$ penalty and do not perform feature selection. As discussed in [16], for high dimensional classification, taking all features into consideration does not work well for two reasons. First, based on prior knowledge, only a small number of variables are relevant to the classification problem: a good classifier in high dimensions should have the ability to sparsely select important variables and discard redundant ones. Second, classifiers using all available variables in high-dimensional settings may have poor classification performance.

Much of the SVM literature has considered variable selection in high-dimensional classification problems to improve performance (e.g., [17,18,19]). Among the DWD literature, to the best of our knowledge, only [16] considered variables selection and classification simultaneously. Wang and Zou [16] considered an $L_{1}$ rather than an $L_{2}$ penalty in problem (2) to improve interpretability through sparsity in the binary classification. Moreover, [16] made selections based on the strengths of input variables within individual classes but ignored the strengths of input variable groupings, thereby selecting more factors than necessary for each class. To overcome this weakness in this paper, we developed a multicategory generalized DWD method that is capable of performing variable selection and classification simultaneously. Our approach incorporates sparsity and group structure information via the sparse group lasso penalty (see [20,21,22,23,24]).

Although DWD is well studied, it is less popular than the SVM for binary classification, arguably for computational and theoretical reasons. For an up-to-date list of works on DWD mostly focused on the q=1 case, see [14,15]. Theoretical asymptotic properties of large-margin classifiers in high dimensional settings were studied in [25], and [26] derived an expression for asymptotic generalization error. In terms of computation, [8] solved the standard DWD problem in (1) as a second-order cone programming (SOCP) problem using a primal-dual interior-point method that is computationally expensive when *N* or *p* is large. To overcome computational bottlenecks, [12] proposed an approach based on a novel formulation of the primal DWD model in (1): this method, proposed in [12], does not scale to large data sets and requires further work. Lam et al. [27] designed a new algorithm for large DWD problems with q≥2 and K=2 based on convergent multi-block ADMM-type methods (see [28]). Wang and Zou [16] solved the lasso-penalized binary DWD problem by combining majorization–minimization and coordinate descent methods: the lasso penalty does not directly permit a SOCP solution. In fact, solution identifiability in the generalized DWD problem with q>1 requires more constraints and remains an open research problem (see [8]). To the best of our knowledge, no work focusing on computational aspects of lasso penalized multicategory generalized DWD (MgDWD) exists. The same holds for sparse group lasso-penalized MgDWD.

The theoretical and computational contributions of this paper are as follows. First, we establish the uniqueness of the minimizer in the population form of the MgDWD problem. Second, we prove a non-asymptotic $L_{2}$ estimation error bound for the sparse group lasso-regularized MgDWD loss function in the ultra-high dimensional setting under mild regularity conditions. Third, we develop a fast, efficient algorithm able to solve the sparse group lasso-penalized MgDWD problem using proximal methods.

The rest of this paper is organized as follows. In Section 2.1, we introduce the MgDWD problem with sparse group lasso penalty. In Section 2.2 and Section 2.3, we establish theoretical properties of the population classifier and regularized empirical loss. We propose a computational algorithm in Section 2.4. Section 3 illustrates the finite sample performance of our method through simulation studies and a real data analysis. Proofs for major theorems are given in the Appendix A.

## 2. Methodology

### 2.1. Model Setup

We begin with some basic set-up and notation. Consider the multicategory classification problem for a random sample ${\left\{\left(y_{i},X_{i}\right)\right\}}_{i=1}^{N}$ of *N* independent and identically distributed (i.i.d.) observations from some distribution P(y,X). Here, *y* is the categorical response taking values in Y={1,…,K}, and $X={\left(x_{1},\ldots ,x_{p}\right)}^{⊤}\in X\subset R^{p}$ is the covariate vector. We wish to obtain a proper separating hyperplane $\left\{X\in X|a_{k}+X^{⊤}b_{k}=0\right\}$ for each category k∈Y, where $a_{k}$ and $b_{k}={\left(b_{1k},\ldots ,b_{pk}\right)}^{⊤}$ are intercept and slope parameters, respectively.

In this paper, we consider MgDWD with sparse group lasso regularization. That is, we estimate a classification boundary by solving the constrained optimization problem

(5) $$\begin{array}{c} \min_{a_{k},\;b_{k}}\frac{1}{N}\sum_{i=1}^{N}\phi_{q}\left(a_{y_{i}}+X_{i}^{⊤}b_{y_{i}}\right)+\lambda_{1}\sum_{k=1}^{K}\sum_{j=1}^{p}\left|b_{jk}\right|+\lambda_{2}\sum_{j=1}^{p}\sqrt{\sum_{k=1}^{K}b_{jk}^{2}}\\ s.t.\sum_{k=1}^{K}a_{k}=0;\;\sum_{k=1}^{K}b_{jk}=0,\;\forall j=1,\ldots ,p, \end{array}$$

where $\phi_{q}$ is as defined in (3).

To approach this problem, we apply the concept of a “margin vector” to extend the definition of a (binary) margin to the multicategory case. Denote the margin vector of an observation $X_{i}$ as $F_{i}={\left(f_{i1},\ldots ,f_{iK}\right)}^{⊤}$, with $f_{ik}=a_{k}+X_{i}^{⊤}b_{k}$ satisfying $\sum_{k=1}^{K}f_{ik}=0$. Let $E_{i}={\left(e_{i1},\ldots ,e_{iK}\right)}^{⊤}$ be the class indicator vector with $e_{ik}=𝟙\left\{y_{i}=k\right\}$. The multicategory margin of the data point $\left(y_{i},X_{i}\right)$ is then given as $f_{iy_{i}}=a_{y_{i}}+X_{i}^{⊤}b_{y_{i}}=E_{i}^{⊤}F_{i}$. Therefore, the MgDWD loss can be rewritten as

(6) $$\begin{array}{c} \phi_{q}\left(a_{y_{i}}+X_{i}^{⊤}b_{y_{i}}\right)=\phi_{q}\left(E_{i}^{⊤}F_{i}\right)=E_{i}^{⊤}\phi_{q}\left(F_{i}\right)=\sum_{k=1}^{K}𝟙\left\{y_{i}=k\right\}\phi_{q}\left(a_{k}+X_{i}^{⊤}b_{k}\right). \end{array}$$

Based on (6), Lemma 1 describes the Fisher consistency of the MgDWD loss.

**Lemma** **1.** *Given X=u, the minimizer of the conditional expectation of (6) is $\tilde{F}\left(u\right)={\left({\tilde{f}}_{1}\left(u\right),\ldots ,{\tilde{f}}_{K}\left(u\right)\right)}^{⊤}$, satisfying*

$$\begin{array}{c} \underset{k\in Y}{\mathrm{argmax}}{\tilde{f}}_{k}\left(u\right)=\underset{k\in Y}{\mathrm{argmax}}\mathrm{Pr}\left\{y=k|X=u\right\}, \end{array}$$

*where*

$$\begin{array}{c} {\tilde{f}}_{k}\left(u\right)=\left\{\begin{array}{cc} Q\sqrt[{q}]{\frac{\mathrm{Pr}\{y=k|X=u\}}{\mathrm{Pr}\{y=k_{*}|X=u\}}},\; & k\neq k_{*}\\ -Q\sum_{l\neq k_{*}}\sqrt[{q}]{\frac{\mathrm{Pr}\{y=l|X=u\}}{\mathrm{Pr}\{y=k_{*}|X=u\}}},\; & k=k_{*}. \end{array}\right. \end{array}$$

*and $k_{*}=\underset{k\in Y}{\mathrm{argmin}}\mathrm{Pr}\left\{y=k|X=u\right\}$.*

Consequently, ${\tilde{f}}_{k}\left(u\right)$ can be treated as an effective proxy of Pr{y=k|X=u} and, for any new observation $X_{*}$, a reasonable prediction of its label $y_{*}$ is

$$\begin{array}{c} {\hat{y}}_{*}=\underset{k\in Y}{\mathrm{argmax}}\left\{a_{k}+X_{*}^{⊤}b_{k}\right\}. \end{array}$$

Speaking to the sparse group lasso (SGL) regularization in (5), the $L_{1}$ penalty encourages an element-wise sparse estimator that selects important variables for each category, indicated by ${\hat{b}}_{jk}\neq 0$. Assuming that parameters in different categories share the same information, we use an $L_{2}$ penalty to encourage a group-wise sparsity structure that removes covariates that are irrelevant across all categories, that is, where ${\hat{\beta }}_{j}={\left(b_{1j},\ldots ,b_{Kj}\right)}^{⊤}=0$. Specifically, let $x_{j}={\left(x_{1j},\cdots ,x_{Nj}\right)}^{T}$ and $B=\left(b_{jk}\right)\in R_{jk}^{p\times K}$, where the *k*-th column $b_{k}$ is the slope vector for the category label *k* and the *j*-th row $\beta_{j}^{⊤}$ is the group coefficient for the variable $x_{j}$. If $x_{j}$ is noise in the classification problem or is not relevant to category label *k*, then the entry $b_{jk}$ of B should be shrunk to exactly zero. The SGL penalty of (5) can be written as a convex combination of the lasso and group lasso penalties in terms of $\beta_{j}$ as

(7) $$\begin{array}{c} \lambda_{1}\sum_{k=1}^{K}\sum_{j=1}^{p}\left|b_{jk}\right|+\lambda_{2}\sum_{j=1}^{p}\sqrt{\sum_{k=1}^{K}b_{jk}^{2}}=\lambda \sum_{j=1}^{p}\left\{\tau {∥\beta_{j}∥}_{1}+\left(1-\tau \right){∥\beta_{j}∥}_{2}\right\}, \end{array}$$

where λ>0 is the scale of the penalty and τ∈[0,1] tunes the propensity between the element-wise and group-wise sparsity structure.

### 2.2. Population MgDWD

In this subsection, some basic results pertaining to unpenalized population MgDWD are given. These results are necessary for further theoretical analysis.

Denote the marginal probability mass of *y* as $\mathrm{Pr}\left(y=k\right)=\pi_{k}$ with $\pi_{k}>0$ and $\sum_{k=1}^{K}\pi_{k}=1$, and the conditional probability density functions of X given y=k by $g\left(X|y=k\right)=g_{k}\left(X\right)$. Let $\Theta =(\theta_{1},\ldots ,\theta_{K})$ be the collection of coefficient vectors $\theta_{k}={\left(a_{k},b_{k}^{⊤}\right)}^{⊤}$ for all labels and $Z={\left(1,X^{⊤}\right)}^{⊤}$. The population version of the MgDWD problem in (6) is

(8) $$\begin{array}{c} L\left(\vartheta \right)=E\left\{I{\left(Y\right)}^{⊤}\phi_{q}\left(\Theta^{⊤}Z\right)\right\}=\sum_{k=1}^{K}\pi_{k}\int_{X}\phi_{q}\left(Z^{⊤}\theta_{k}\right)g_{k}\left(x\right)dx, \end{array}$$

where ϑ=vec{Θ} is the vectorization of the matrix Θ and $I\left(Y\right)={\left(𝟙\left\{y=1\right\},\ldots ,𝟙\left\{y=K\right\}\right)}^{⊤}$ is a random vector. Denote the true parameter value $\vartheta^{*}$ as a minimizer of the population MgDWD problem, namely,

$$\begin{array}{c} \vartheta^{*}\in \underset{\vartheta \in C}{\mathrm{argmin}}L\left(\vartheta \right), \end{array}$$

where $C=\left\{\vartheta \in R^{K(p+1)}|C\vartheta =0_{K}\right\}$ is the set of sum-constrained ϑ with $C=1_{K}^{⊤}⊗I_{p+1}$, where ⊗ denotes the Kronecker product.

To facilitate our theoretical analysis, we first define the gradient vector and Hessian matrix of the population MgDWD loss function. We then introduce some regularity conditions necessary to derive theoretical properties of this problem. Let diag{v} be a diagonal matrix constructed from the vector v, and let ∘ and ⊕ be the Hadamard product and the direct matrix sum, respectively. Denote the gradient vector of the population MgDWD loss function (8) as

$$\begin{array}{c} S\left(\vartheta \right)=E\left(\{I\left(Y\right)\circ \phi_{q}^{\prime }\left(\Theta^{⊤}Z\right)\}⊗Z\right)=\mathrm{vec}\left(S_{1},\cdots ,S_{K}\right), \end{array}$$

with

$$\begin{array}{c} S_{k}=E\left\{𝟙\left\{y=k\right\}\phi_{q}^{\prime }\left(Z^{⊤}\theta_{k}\right)Z\right\}=\pi_{k}\int_{X}\phi_{q}^{\prime }\left(Z^{⊤}\theta_{k}\right)Zg_{k}\left(X\right)dX, \end{array}$$

and its Hessian matrix as

$$\begin{array}{c} H\left(\vartheta \right)=E\left\{\mathrm{diag}\left\{I\left(Y,\;X\right)\circ \varphi_{q}^{\prime \prime }\left(\Theta^{⊤}Z\right)\right\}⊗\left({\mathrm{ZZ}}^{⊤}\right)\right\}=\overset{K}{\underset{k=1}{⨁}}H_{k}, \end{array}$$

where $\varphi_{q}^{\prime \prime }$ denotes the second derivative of the function $\varphi_{q}$; I(Y,X)=I(Y)∘I(X) is a random vector with $I\left(X\right)={\left(𝟙\left\{X\in X_{1}\right\},\ldots ,𝟙\left\{X\in X_{k}\right\}\right)}^{⊤}$ and $X_{k}=\left\{X\in X|Z^{⊤}\theta_{k}>Q\right\}$; and

$$\begin{array}{c} H_{k}=E\left\{𝟙\left\{y=k,\;X\in X_{k}\right\}\varphi_{q}^{\prime \prime }\left(Z^{⊤}\theta_{k}\right){\mathrm{ZZ}}^{⊤}\right\}=\pi_{k}\int_{X_{k}}\varphi_{q}^{\prime \prime }\left(Z^{⊤}\theta_{k}\right){\mathrm{ZZ}}^{⊤}g_{k}\left(X\right)dX. \end{array}$$

The block structure of H(ϑ) implies a parallel relationship between each category. The relationship between the $\theta_{k}$ is reflected by the sum-to-zero constraint in the definition of C.

We assume the following regularity conditions.

(C1) The densities of X given y=k∈Y, i.e., the $g_{k}\left(X\right)$, are continuous and have finite second moments.

(C2) $0<\mathrm{Pr}\{X\in X_{k}^{*}|y=k\}<1$ for all k∈Y, where $X_{k}^{*}=\left\{X\in X|Z^{⊤}\theta_{k}^{*}>Q\right\}$.

(C3) $\mathrm{Var}\left\{X|X\in X_{k}^{*},\;y=k\right\}≻O_{p}$ for all k∈Y.

**Remark** **1.** *Condition (C1) ensures that L, S and H are well defined and continuous in ϑ. For the theoretically optimal hyperplane $\left\{X\in X|Z^{⊤}\theta_{k}^{*}=0\right\}$, the case with $\theta_{k}^{*}=0_{p+1}$ leaves X useless for classification. On the other hand, when $a_{k}^{*}\neq 0$ and $b_{k}^{*}=0_{p}$, the hyperplane is the empty set and is similarly meaningless. Condition (C2) is proposed to avoid the case where $b_{k}^{*}=0_{p}$ so that $\vartheta^{*}$ always contains information relevant to the classification problem. For bounded random variables, condition (C2) should be assumed with caution. Condition (C3) implies the positive definiteness of $H(\vartheta^{*})$.*

By convexity and the second-order Lagrange condition, the following theorem shows that the local minimizer of the population MgDWD problem exists and is unique.

**Theorem** **1.** *Under the regularity conditions (C1)–(C3), the true parameter $\vartheta^{*}\in C$ is the unique minimizer of L(ϑ) with $b_{k}^{*}\neq 0_{p}$, and*

$$\begin{array}{c} L\left(\vartheta^{*}\right)=\sum_{k=1}^{K}A\left(k,q\right)\pi_{k}, \end{array}$$

*with 0≤u(k,q)≤A(k,q)≤v(k,q)≤1, where*

$$\begin{array}{c} A\left(k,q\right)=1-E\left\{𝟙\left\{X\in X_{k}^{*}\right\}\left\{1-{\left(\frac{Q}{Z^{⊤}\theta_{k}^{*}}\right)}^{q}\right\}|y=k\right\},\\ u\left(k,q\right)=\mathrm{Pr}\left\{X\notin X_{k}^{*}|y=k\right\}+Q^{2q}\mathrm{Pr}\left\{Q<Z^{⊤}\theta_{k}^{*}\leq Q^{-1}|y=k\right\},\\ v\left(k,q\right)=\mathrm{Pr}\left\{Z^{⊤}\theta_{k}^{*}\leq 1|y=k\right\}+\underset{\epsilon >0}{\inf}{\left(\frac{Q}{1+\epsilon }\right)}^{q}\mathrm{Pr}\left\{Z^{⊤}\theta_{k}^{*}>1+\epsilon |y=k\right\}. \end{array}$$

The bounds in Theorem 1 show how *q* affects the loss function $L(\vartheta^{*})$. The upper bound v(k,q) is a decreasing function of *q* with

$$\begin{array}{c} \lim_{q\to 0}v\left(k,q\right)=1\;\mathrm{and}\;\lim_{q\to \infty }v\left(k,q\right)=\mathrm{Pr}\left\{Z^{⊤}\theta_{k}^{*}\leq 1|y=k\right\}. \end{array}$$

In the lower bound u(k,q), the first term $\mathrm{Pr}\left\{X\notin X_{k}^{*}|y=k\right\}$ is an increasing function of *q* and the last term $Q^{2q}\mathrm{Pr}\left\{Q<Z^{⊤}\theta_{k}^{*}\leq Q^{-1}|y=k\right\}$ is a decreasing function of *q*, with

$$\begin{array}{c} \lim_{q\to 0}u\left(k,q\right)=1\;\mathrm{and}\;\lim_{q\to \infty }u\left(k,q\right)=\mathrm{Pr}\left\{Z^{⊤}\theta_{k}^{*}\leq 1|y=k\right\}. \end{array}$$

Consequently, for the given population P(y,X), a larger *q* encourages the population MgDWD estimator to focus more on the regions $\left\{X\notin X_{k},\;y=k\right\}$ that correspond to misclassifications. As a result, the estimator’s performance will be similar to the hinge loss as q→∞. Setting *q* too small will lead to an ineffective classifier due to the unreasonable penalty placed on the well classified region $\left\{X\in X_{k},\;y=k\right\}$. This variation in the lower bound with respect to *q* provides a necessary condition for the existence of an optimal *q*.

**Remark** **2.** *The explicit relationship between q and $\vartheta^{*}$ is complicated. While it may be more desirable to prove that a greater value of q results in a smaller value of the loss function L(ϑ), there is no explicit formula for the optimal value $\vartheta^{*}$ in terms of q.*

### 2.3. Estimator Consistency

Under the unpenalized framework presented in the previous subsection, all covariates will contribute to the classification task for each category: this scenario may lead to a classifier that overfits to the training data set. In this subsection, we study the consistency of the estimator for (5) in ultra-high dimensional settings.

To achieve structural sparsity in the estimator, the regularization parameter λ in (7) must be large enough to dominate the gradient of the empirical MgDWD loss evaluated at the theoretical minimizer $\vartheta^{*}=\mathrm{vec}\left\{\Theta^{*}\right\}$ with high probability. On the other hand, λ should also be as small as possible to reduce the bias incurred by the SGL regularization term

$$\begin{array}{c} P\left(\beta \right)=\sum_{j=1}^{p}\tau {∥\beta_{j}∥}_{1}+\left(1-\tau \right){∥\beta_{j}∥}_{2}. \end{array}$$

Lemma 2 provides a suitable choice of λ under the following assumption.

(A1) The predictors $X=\left(x_{1},\ldots ,x_{p}\right)\in R^{p}$ are independent sub-Gaussian random vectors satisfying $EX=0_{p}$, and where Var(X)=Σ, there exists a constant κ>0 such that for any $\gamma \in R^{p}$, $E\exp\left(\gamma^{⊤}\Sigma^{-1/2}X\right)\leq \exp\left({∥\gamma ∥}_{2}^{2}\kappa^{2}/2\right)$. From here on, we define $\varsigma_{1}^{2}$ as the largest eigenvalue of Σ.

**Lemma** **2.** *Denote $S\left(\vartheta^{*}\right)=\left(I_{K}⊗Z^{⊤}\right)\mathrm{diag}\left(\mathrm{vec}\left\{E\right\}\right)\mathrm{vec}\left\{\phi_{q}^{\prime }\left(Z\Theta^{*}\right)\right\}$, where $E={\left(E_{1},\ldots ,E_{N}\right)}^{⊤}$, $Z={\left(Z_{1},\ldots ,Z_{N}\right)}^{⊤}$ with $Z_{i}={\left(1,X_{i}^{⊤}\right)}^{⊤}$, and $I_{K}$ is the identity matrix of size K. Under condition (A1),*

$$\begin{array}{c} \tilde{P}\left\{PS(\vartheta^{*})\right\}\leq \tau \Lambda_{1}+\left(1-\tau \right)\Lambda_{2} \end{array}$$

*with probability at least $1-2{\left(Kp\right)}^{1-c_{1}^{2}}-p^{1-c_{2}^{2}}$, where*

$$\begin{array}{c} P=\left(I_{K}-K^{-1}1_{K}1_{K}^{⊤}\right)⊗I_{p+1},\\ \Lambda_{1}=\max\left\{\varsigma_{1}\kappa ,1\right\}\left(1-\frac{1}{K}\right)\sqrt{\frac{2\log(pK)}{N}},\\ \Lambda_{2}=\max\left\{2\sqrt{2}\varsigma_{1}\kappa ,1\right\}\left\{c_{2}\sqrt{\left(1-\frac{1}{K}\right)\frac{2\log(p)}{N}}+\sqrt{\frac{K-1}{N}}\right\}, \end{array}$$

*for constants $c_{1},c_{2}>1$.*

It is difficult to obtain a closed form for the conjugate of the SGL penalty, say, $\bar{P}\left(v\right)=\sup_{u\in C\backslash \{0\}}\frac{\langle u,v\rangle }{P(u)}$. Instead, we use a regularized upper bound $\tilde{P}\left(v\right)\geq \bar{P}\left(v\right)$. Based on Lemma 2, we propose a theoretical tuning parameter value

(9) $$\begin{array}{c} \lambda =c_{0}\sqrt{\frac{\log(pK)}{N}}, \end{array}$$

where $c_{0}$ is some given constant satisfying $\lambda >\tau \Lambda_{1}+\left(1-\tau \right)\Lambda_{2}$.

Before we can derive an error bound for the estimator in (5), we impose two additional assumptions.

(A2) For the true parameter value $\vartheta^{*}$, there is a $\left(s_{e},s_{g}\right)$-sparse structure in the coefficients $B^{*}$ with element-wise and group-wise support sets

$$\begin{array}{c} E=\left\{\left(j,k\right)\in \left\{1,\ldots ,p\right\}\times \left\{1,\ldots ,K\right\}|b_{jk}^{*}\neq 0\right\}\;\mathrm{and}\;G=\left\{j\in \left\{1,\ldots ,p\right\}|\beta_{j}^{*}\neq 0_{K}\right\} \end{array}$$

with cardinality $\left|E\right|=s_{e}$ and $\left|G\right|=s_{g}$, respectively.

(A3) There exist some positive constants $\varsigma_{2}$ and $\varsigma_{3}$ such that

$$\begin{array}{c} \varsigma_{2}^{2}=\max_{\gamma \in V}\frac{∥\mathrm{diag}\left\{\mathrm{vec}\left(E^{⊤}\right)\right\}\left(Z⊗I_{K}\right){\gamma ∥}_{2}^{2}}{{N∥\gamma ∥}_{2}^{2}}\;\mathrm{and}\;\varsigma_{3}^{2}=\min_{\gamma \in U}\frac{\gamma^{⊤}H\left(\vartheta^{*}\right)\gamma }{\gamma^{⊤}\gamma } \end{array}$$

with $V=\left\{v\in R^{K(p+1)}{|0<∥v∥}_{0}\leq s_{e}+K\right\}$ and

$$\begin{array}{c} U=\left\{\delta \in R^{K(p+1)}|\frac{\tau }{1-\tau }{∥\delta_{E_{+}}∥}_{1}+\sum_{j\in G_{+}}{∥\delta_{j}∥}_{2}\geq C_{0}\left(\frac{\tau }{1-\tau }{∥\delta_{E^{c}}∥}_{1}+\sum_{j\notin G}{∥\delta_{j}∥}_{2}\right)\right\}, \end{array}$$

where $C_{0}=\frac{\left(c_{0}-1\right)}{\left(c_{0}+1\right)}$, $E^{c}$ is the complement of E, $E_{+}=E\cup \left\{l=1+\left(k-1\right)\left(p+1\right)|k=1,\ldots ,K\right\}$, and $G_{+}=G\cup \left\{0\right\}$.

Under the choice of λ given in (9), we show the $L_{2}$-consistency of the estimator in (5).

**Theorem** **2.** *Suppose that conditions (A1)–(A3) hold. Then with $\lambda =c_{0}\sqrt{\frac{\log(pK)}{N}}$ in (5), we have that*

$$\begin{array}{c} ∥\hat{\vartheta }-\vartheta^{*}{∥}_{2}\leq \left\{C_{1}\sqrt{s_{e}+K}+C_{2}\sqrt{s_{g}+1}\right\}\sqrt{\frac{\log(pK)}{N}} \end{array}$$

*with probability at least $1-2{\left(Kp\right)}^{2\left(s_{e}+K\right)\left(1-c_{3}^{2}\right)}$, where $C_{1}=2\varsigma_{3}^{-2}\left\{c_{0}\tau +\left(\sqrt{2}+2c_{3}\right)\varsigma_{2}\right\}$ and $C_{2}=2\varsigma_{3}^{-2}c_{0}\left(1-\tau \right)$.*

**Remark** **3.** *The sub-Gaussian distribution assumption (A1) is common in high-dimensional scenarios. This assumption characterizes the tail behavior of a collection of random variables including Gaussian, Bernoulli, and bounded variables as special cases. Assumption (A2) describes structural sparsity at two levels. The element-wise size $s_{e}<p$ is the size of the underlying generative model, and the group-wise size $s_{g}<pK$ is the size of the signal covariate set. Both $s_{e}$ and $s_{g}$ are allowed to depend on the sample size N. As a result, the dimension p is allowed to increase with the sample size N. Assumption (A3) guarantees that eigenvalues are positive in this sparse scenario.*

**Remark** **4.** *In practice, the tuning parameters λ and τ in (7) are commonly chosen by M-fold cross validation. That is, we choose the pair (τ,λ) with the highest prediction accuracy among the sub-data sets $D_{m}$, specifically,*

$$\begin{array}{c} CV\left(\tau ,\lambda \right)=\sum_{m=1}^{M}\sum_{i\in D_{m}},𝟙\left\{y_{i}={\hat{y}}_{i}\left(\tau ,\lambda \right)\right\} \end{array}$$

*where ${\hat{y}}_{i}\left(\tau ,\lambda \right)=\underset{k\in Y}{\mathrm{argmax}}Z_{i}^{⊤}{\hat{\theta }}_{k}\left(\tau ,\lambda \right)$.*

### 2.4. Computational Algorithm

In this section, we propose an efficient algorithm to solve problem (5). Our approach uses the proximal algorithm (see [29]) for solving high dimensional regularization problems. In two main steps, this approach obtains a solution to the constrained optimization problem by applying the proximal operator to the solution to the unconstrained problem.

Since regularization is not needed for the intercept terms $\alpha ={\left(a_{1},\ldots ,a_{K}\right)}^{⊤}$, it can be separated from the coefficients in B. The empirical MgDWD loss of (8) is given by

$$\begin{array}{c} L\left(\vartheta \right)=\frac{1}{N}\sum_{i=1}^{N}E_{i}^{⊤}\phi_{q}\left(F_{i}\right)=\frac{1}{N}\mathrm{tr}\left\{E\phi_{q}\left(F^{⊤}\right)\right\}=\frac{1}{N}\mathrm{vec}{\left\{E^{⊤}\right\}}^{⊤}\mathrm{vec}\left\{\phi_{q}\left(F^{⊤}\right)\right\} \end{array}$$

where $F={\left(f_{ik}\right)}_{N\times K}=Z\Theta =1_{N}\alpha^{⊤}+\mathrm{XB}$. Various properties of the loss function L(ϑ) follow below.

**Lemma** **3.** *The loss function L(ϑ) has Lipschitz continuous partial derivatives. In particular, for $S\left(\alpha \right)=\frac{\partial L(\theta )}{\partial \alpha }=\frac{1}{N}{\left\{E\circ \phi_{q}^{\prime }\left(F\right)\right\}}^{⊤}1_{N}$ and any $u,v\in R^{K}$, we have that*

$$\begin{array}{c} ∥S\left(u\right)-S\left(v\right){∥}_{2}\leq \sqrt{\frac{n_{\max}}{N}}\frac{{\left(q+1\right)}^{2}}{q}{∥u-v∥}_{2}, \end{array}$$

*where $n_{\max}$ is the largest group sample size. For $S\left(B\right)=\frac{\partial L(\theta )}{\partial B}=\frac{1}{N}{\left\{E\circ \phi_{q}^{\prime }\left(F\right)\right\}}^{⊤}X$ and any $U,V\in R^{p\times K}$, we have that*

$$\begin{array}{c} ∥\mathrm{vec}\left\{S\left(U\right)-S\left(V\right)\right\}{∥}_{2}\leq \frac{\max_{k}{∥\mathrm{diag}\left(e_{k}\right)X∥}_{2}^{2}}{N}\frac{{\left(q+1\right)}^{2}}{q}{∥\mathrm{vec}\left\{U-V\right\}∥}_{2}, \end{array}$$

*where $e_{k}$ is the k-th column of E and indicates the observations belonging to the k-th group.*

Hence, following the majorization–minimization scheme, we can majorize the empirical MgDWD loss L(ϑ) by a quadratic function, that is,

$$\begin{array}{cc} L(\vartheta )\leq & L\left(\vartheta_{*}\right)+S{\left(\alpha_{*}\right)}^{⊤}\left(\alpha -\alpha_{*}\right)+\frac{L_{\alpha }}{2}{∥\alpha -\alpha_{*}∥}_{2}^{2}\\ \; & +\mathrm{vec}{\left\{S\left(B\right)\right\}}^{⊤}\mathrm{vec}\left\{B-B_{*}\right\}+\frac{L_{B_{*}}}{2}{∥\mathrm{vec}\left\{B-B_{*}\right\}∥}_{2}^{2}, \end{array}$$

for some $\vartheta_{*}=\mathrm{vec}\left\{{\left(\alpha_{*},B_{*}^{⊤}\right)}^{⊤}\right\}$, where $L_{\alpha }$ and $L_{B}$ denote the Lipschitz constants in Lemma 3. Instead of minimizing L(ϑ) directly, we apply gradient descent to minimize its surrogate upper bound function. The gradient descent updates are given by

(10) $$\begin{array}{c} \alpha_{*}=\alpha -\frac{q{\left(q+1\right)}^{-2}}{\sqrt{n_{\max}N}}{\left\{E\circ \phi_{q}^{\prime }\left(F\right)\right\}}^{⊤}1_{N}, \end{array}$$

(11) $$\begin{array}{c} B_{*}=B-\frac{q{\left(q+1\right)}^{-2}}{\max_{k}{∥\mathrm{diag}\left(e_{k}\right)X∥}_{2}^{2}}{\left\{E\circ \phi_{q}^{\prime }\left(F\right)\right\}}^{⊤}X. \end{array}$$

Next, we address the problem’s constraints and regularization simultaneously by applying the proximal operator. For $\alpha_{*}$, it is clear that

(12) $$\begin{array}{c} \alpha_{\mathrm{new}}=\underset{\alpha^{⊤}1_{K}=0}{\mathrm{argmin}}{∥\alpha -\alpha_{*}∥}_{2}^{2}=P_{K}\alpha_{*}, \end{array}$$

where $P_{K}=I_{K}-k^{-1}1_{K}1_{K}$. For $B_{*}={\left(\beta_{1*},\ldots ,\beta_{p*}\right)}^{⊤}$, the minimization problem can be expressed as

(13) $$\begin{array}{cc} B_{\mathrm{new}}= & \underset{B1_{K}=0_{p}}{\mathrm{argmin}}\frac{1}{2}∥\mathrm{vec}\left\{B-B_{*}\right\}{∥}_{2}^{2}+\frac{\lambda_{1}}{L_{B}}∥\mathrm{vec}\left\{B\right\}{∥}_{1}+\frac{\lambda_{2}}{L_{B}}{∥\mathrm{vec}\left\{B\right\}∥}_{1,2}\\ = & \underset{B1_{K}=0_{p}}{\mathrm{argmin}}\sum_{j=1}^{p}\frac{1}{2}∥\beta_{j}-\beta_{j*}{∥}_{2}^{2}+\frac{\lambda_{1}}{L_{B}}∥\beta_{j}{∥}_{1}+\frac{\lambda_{2}}{L_{B}}{∥\beta_{j}∥}_{2}, \end{array}$$

which implies that we can implement minimization for *p* groups in parallel. The following theorem provides the solution to (13).

**Theorem** **3.** *Let $\rho_{1},\;\rho_{2}\geq 0$ and $\beta_{*}\in R^{K}$. Then the constrained regularization problem*

$$\begin{array}{c} \min_{\beta \in R^{K}}\frac{1}{2}∥\beta -\beta_{*}{∥}_{2}^{2}+\rho_{1}{∥\beta ∥}_{1}+\rho_{2}{∥\beta ∥}_{2}\\ s.t.\;\;\beta^{⊤}1_{K}=0 \end{array}$$

*has a solution of the form*

(14) $$\begin{array}{c} \beta^{*}={\left\{1-\frac{\rho_{2}}{∥P_{K}\left(\beta_{*}-\rho_{1}u\right){∥}_{2}}\right\}}_{+}P_{K}\left(\beta_{*}-\rho_{1}u\right) \end{array}$$

*for some $u\in {\partial ∥\beta ∥}_{1}$.*

In the special case with $\rho_{2}=0$, the constrained regularization problem in Theorem 3 reduces to the constrained lasso problem with solution ${\tilde{\beta }}^{*}=P_{K}\left(\beta_{*}-\rho_{1}u\right)$. Combined with (14), the proximal operator U, given by

(15) $$\begin{array}{c} \beta^{*}=U\left({\tilde{\beta }}^{*},\rho_{2}\right)={\left\{1-\frac{\rho_{2}}{∥{\tilde{\beta }}^{*}{∥}_{2}}\right\}}_{+}{\tilde{\beta }}^{*}, \end{array}$$

can be introduced to realize the group sparsity of ${\tilde{\beta }}^{*}$.

For the standard lasso problem, the subgradient u has a closed form given by ${\tilde{\beta }}^{*}=\beta_{*}-\rho_{1}u=S\left(\beta_{*},\rho_{1}\right)$, with $S\left(u,v\right)=\mathrm{sign}\left(u\right){\left(|u|-v\right)}_{+}$. However, under the constraint on ${\tilde{\beta }}^{*}$, the naive solution $P_{K}S\left(\beta_{*},\rho_{1}\right)$ is misleading in that it satisfies the constraint but does not achieve shrinkage, let alone loss function minimization. The term $P_{K}u$ is suggestive of the intersection between the subdifferential set ${\partial ∥\beta ∥}_{1}$ and the constraint set $\left\{\beta \in R^{K}|\beta^{⊤}1_{K}=0\right\}$; in this sense, ${\tilde{\beta }}^{*}$ might not have a closed form. Here we consider using coordinate descent to solve the constrained lasso problem. For some fixed coordinate *m*, since $\beta^{⊤}1_{K}=0$, we have that $b_{m}=-\sum_{l\neq m}b_{l}$. Rewriting the objective function of the lasso-constrained problem in a coordinate-wise form, we obtain

(16) $$\begin{array}{cc} \sum_{l=1}^{K}\frac{1}{2}{\left(b_{l}-b_{l*}\right)}^{2}+\rho_{1}\left|b_{l}\right|= & {\left(b_{k}-\frac{\left(b_{k*}-b_{m*}\right)}{2}+\frac{1}{2}\sum_{l\neq k,m}^{K}b_{l}\right)}^{2}+\rho_{1}\left\{|b_{k}|+|b_{k}+\sum_{l\neq k,m}^{K}b_{l}|\right\}\\ \; & +\frac{1}{4}{\left(b_{k*}+b_{m*}+\sum_{l\neq k,m}^{K}b_{l}\right)}^{2}+\sum_{l\neq k,m}^{K}\frac{1}{2}{\left(b_{l}-b_{l*}\right)}^{2}+\rho_{1}\left|b_{l}\right|. \end{array}$$

Next, Theorem 4 provides the solution to the optimization problem (16).

**Theorem** **4.** *Suppose that t,s∈R and ϱ≥0. Then the regularization problem*

$$\begin{array}{c} \min_{b\in R}\frac{1}{2}{\left(b-t\right)}^{2}+\varrho \left\{|b|+|b+s|\right\} \end{array}$$

*has solution*

$$\begin{array}{cc} b^{*}= & \left\{\begin{array}{cc} t, & \left|t|<C(s,t\right)\\ -C(s,t), & C(s,t)\leq |t|\leq C(s,t)+2\varrho \\ \mathrm{sign}(t)(|t|-2\varrho ), & |t|>C(s,t)+2\varrho \end{array}\right.\\ =\; & t-S\left(t,C(s,t)\right)+S\left\{S\left(t,C(s,t)\right),2\varrho \right\}, \end{array}$$

*where $C\left(s,t\right)=\frac{1-\mathrm{sign}(s)\mathrm{sign}(t)}{2}\left|s\right|$.*

By Theorem 4, given some m∈{1,…,K}, the coordinate-wise minimizer for any k≠m can be expressed as the proximal operator

(17) $$\begin{array}{c} b_{k}=T\left(t,s,\rho_{1}\right)=t-S\left(t,C(s,t)\right)+S\left\{S\left(t,C(s,t)\right),\rho_{1}\right\}, \end{array}$$

with $s=\sum_{l\neq k,m}b_{l}$ and $t=(b_{k*}-b_{m*}-s)/2$. If we fix *m* during iteration, then the shrinkage of $b_{m}$ will be indirectly reflected in the other $b_{k}$. We propose that *m* change with *k* in the coordinate-wise minimization process to ensure that every coordinate can be equally shrunk. We summarize our proposed algorithm in Algorithm 1.
**Algorithm 1:** Proximal gradient descent algorithm for SGL-MgDWD.**Input:**$\lambda_{1},\;\lambda_{2}.$**Initialization:**$\alpha^{\left(0\right)}=0_{K}$, $B^{\left(0\right)}=O_{p\times K}$, l=0.1:**repeat**2:    Update α according to (10) and (12):
$$\begin{array}{c} \alpha^{\left(l+1\right)}=P_{K}\left\{\alpha^{\left(l\right)}-L_{\alpha }^{-1}S\left(\alpha^{\left(l\right)}\right)\right\}. \end{array}$$3:    Update $\tilde{B}$ according to (11):
$$\begin{array}{c} \tilde{B}=B^{\left(l\right)}-L_{B}^{-1}S\left(B^{\left(l\right)}\right). \end{array}$$4:    Set $B^{\left(l+1\right)}\leftarrow \tilde{B}$.5:    **repeat**6:        **for** m=1 to *K* **do**7:           **for** *k* in {1,…,K}\m **do**8:               Update (t,s):
$$\begin{array}{c} t={\tilde{b}}_{k}-{\tilde{b}}_{m},\;s=\sum_{r=1}^{K}b_{r}^{\left(l+1\right)}-b_{k}^{\left(l+1\right)}-b_{m}^{\left(l+1\right)}. \end{array}$$9:               Update $b_{k}^{\left(l+1\right)}$ according to (17) and $b_{m}^{\left(l+1\right)}$ by constraint:
$$\begin{array}{c} b_{k}^{\left(l+1\right)}=T\left(t,s,L_{B}^{-1}\lambda_{1}\right),\;b_{m}^{\left(l+1\right)}=-s-b_{k}^{\left(l+1\right)}. \end{array}$$10:           **end for**11:        **end for**12:    **until** $B^{\left(l+1\right)}$ convergence.13:    Update $B^{\left(l+1\right)}$ according to (15):
$$\begin{array}{c} B^{\left(l+1\right)}=U\left(B^{\left(l+1\right)},L_{B}^{-1}\lambda_{2}\right). \end{array}$$14:    Set l←l+1.15:**until** some condition is met.**Output:**$\alpha^{\left(l\right)}$ and $B^{\left(l\right)}$.


## 3. Numerical Analysis

In the following section, we use both simulated and real data sets to evaluate the finite sample properties of our proposed method. We compare the finite sample performance of SGL-MgDWD with $L_{1}$-regularized multinomial logistic regression ($L_{1}$-logistic).

### 3.1. Simulation Studies

The data is generated from the following model. Consider the *K*-category classification problem where $\pi_{k}=K^{-1}$ and $g_{k}\left(X\right)$ is the density function of a normal distribution with mean vector $\mu_{k}={\left(\mu_{1k},\mu_{2k},0_{p-2}^{⊤}\right)}^{⊤}$ and covariance matrix $I_{p}$, where $\left(\mu_{1k},\mu_{2k}\right)=\left(2\cos\left(\pi r_{k}\right),\;2\sin\left(\pi r_{k}\right)\right)$ with $r_{k}=\frac{2(k-1)}{K}$, for k=1,…,K. In this model, only the first two variables contribute to the classification and their corresponding parameter vectors $\beta_{1}$ and $\beta_{2}$ form two groups of coefficients. The true model has the sparsity structure $\left(s_{e},s_{g}\right)=\left(2K,2\right)$ for a total of K(p+1) coefficients. We set the sample size for each category to $n_{k}=50,100,200$ and 400, and the number of classes to K=5 and 11. We consider dimensionality p=100 and 1000.

In what follows, we compare the proposed SGL-MgDWD method with the OVR method based on SGL-MgDWD with K=2 (OVR-SGL-gDWD). For SGL-MgDWD, logistic regression and OVR, the tuning parameter λ is optimized over a discrete set by minimizing the prediction error using 5-fold cross validation. In each simulation, we conduct 100 runs and use a testing set of equal size to evaluate each method’s performance using the following criteria:

- Testing set accuracy, measuring the rate of correct classification;
- Signal, as the average number of correctly-selected element-wise and group-wise signals, that is, with ${\hat{b}}_{jk}\neq 0$ and ${\hat{\beta }}_{j}\neq 0$, respectively, denoted by the pair $\left(s_{e}^{+},s_{g}^{+}\right)$;
- Noise, as the average number of incorrectly-selected element-wise and group-wise components, that is, with ${\hat{b}}_{jk}=0$ and ${\hat{\beta }}_{j}=0$, respectively, denoted by the pair $\left(n_{e}^{+},n_{g}^{+}\right)$.

Simulation results are summarized in Table 1 and Table 2.

As shown in Table 1 and Table 2, the proposed SGL-MgDWD method performs better than the $L_{1}$-logistic and OVR methods. Specifically, in each scenario, predictions from the SGL-MgDWD method had higher accuracy relative to the other two methods. Similarly, the SGL-MgDWD method correctly selected the signal components of the model with fewer incorrectly-selected noise components, again relative to the $L_{1}$-logistic and OVR methods. These simulation results also demonstrate that test accuracy increases with increasing sample size $n_{k}$ and that test accuracy decreases with higher dimension *p* at fixed $n_{k}$. This is consistent with the derived theoretical properties. All computations were performed on a Tensorflow 2.3 CPU on Threadripper 2950X at 4.1 Ghz.

### 3.2. HIV Data Analysis

Symptomatic distal sensory polyneuropathy (sDSP) is a common debilitating condition among people with HIV. This condition leads to neuropathic pain and is associated with substantial comorbidities and increased health care costs. Plasma miRNA profiles show differences between HIV patients with and without sDSP, and several miRNA biomarkers are reported to be associated with the presence of sDSP in HIV patients (see [30]). The corresponding binary classification problem was analyzed in [30] using random forest classifiers. However, the HIV data set can be further classified into four classes. The HIV data set has 1715 miRNA measures for 40 patients and is partitioned into four groups (K=4) with $n_{k}=10$ patients each category: non-HIV, HIV with no brain damage (HIVNBD), HIV with brain damage but stable (HIVBDS) and HIV with brain damage and unstable (HIVBDU). In the following analysis, we apply our proposed method to this classification problem. The primary aim was to identify critical miRNA biomarkers for each of the four groups. Beyond achieving a finer classification, this analysis is helpful in assessing related pathogenic effects for each patient group.

Given the small sample size of N=40, we chose the tuning parameter λ by maximizing leave-one-out cross validation accuracy. We fixed (q,τ)=(1,0.1). Table 3 shows the signal for coefficient estimates obtained from the SGL-MgDWD method using the selected λ. We conclude that there are 22 critical miRNA biomarkers important to the classification problem. In particular, the biomarkers miR-25-star, miR-3171, miR-3924 and miR-4307 are not relevant to the non-HIV group; miR-4641, miR-4655-3p and miR-660 are not relevant to the HIVNBD group; miR-217 and miR-4683 are not relevant to the HIVBDS group; and miR-217 and miR-4307 are not relevant to the HIVBDU group.

## Figures and Tables

**Table 1:** Simulation results for the SGL-MgDWD, $L_{1}$-logistic, and OVR methods with K=5. Time is measured relative to a baseline logistic regression model with K=5, p=100, and N=50. Numbers in parentheses denote standard deviations.

$n_{k}$	*p*	Method	Test Accuracy	Signal $\left(s_{e}^{+},s_{g}^{+}\right)$	Noise $\left(n_{e}^{+},n_{g}^{+}\right)$	Time (SD)
50	100	SGL-MgDWD	0.980	(9.99, 2)	(0, 0)	1.150 (0.173)
		$L_{1}$-logistic	0.979	(9.00, 2)	(116.98, 26.17)	1.000 (0.153)
		OVR-SGL-gDWD	0.912	-	-	-
	1000	SGL-MgDWD	0.979	(10, 2)	(6.96, 1.94)	5.290 (0.166)
		$L_{1}$-logistic	0.966	(10, 2)	(2793.65, 722.38)	5.130 (0.063)
		OVR-SGL-gDWD	0.740	-	-	-
100	100	SGL-MgDWD	0.981	(10, 2)	(0.07, 0.03)	1.453 (0.155)
		$L_{1}$-logistic	0.980	(8.82, 2)	(35.18, 3.98)	1.258 (0.127)
		OVR-SGL-gDWD	0.828	-	-	-
	1000	SGL-MgDWD	0.980	(10, 2)	(1.01, 0.25)	4.863 (0.150)
		$L_{1}$-logistic	0.978	(9.93, 2)	(1380.38, 192.37)	4.703 (0.061)
		OVR-SGL-gDWD	0.546	-	-	-
200	100	SGL-MgDWD	0.980	(10, 2)	(7.67, 2.08)	1.776 (0.164)
		$L_{1}$-logistic	0.980	(9.39, 2)	(13.1, 0.72)	1.709 (0.175)
		OVR-SGL-gDWD	0.934	-	-	-
	1000	SGL-MgDWD	0.982	(10, 2)	(1.09, 0.29)	8.641 (0.186)
		$L_{1}$-logistic	0.981	(9.79, 2)	(199.02, 2.51)	2.505 (0.121)
		OVR-SGL-gDWD	0.950	-	-	-
400	100	SGL-MgDWD	0.981	(10, 2)	(0.02, 0)	2.792 (0.159)
		$L_{1}$-logistic	0.981	(10, 2)	(4.72, 3.95)	2.828 (0.115)
		OVR-SGL-gDWD	0.979	-	-	-
	1000	SGL-MgDWD	0.981	(10, 2)	(4.72, 3.95)	15.800 (0.221)
		$L_{1}$-logistic	0.981	(9.6, 2)	(16.17, 0.02)	17.915 (1.585)
		OVR-SGL-gDWD	0.964	-	-	-

**Table 2:** Simulation results for the SGL-MgDWD, $L_{1}$-logistic, and OVR methods with K=11. Time is measured relative to a baseline logistic regression model with K=5, p=100, and N=50. Numbers in parentheses denote standard deviations.

$n_{k}$	*p*	Method	Test Accuracy	Signal $\left(s_{e}^{+},s_{g}^{+}\right)$	Noise $\left(n_{e}^{+},n_{g}^{+}\right)$	Time (SD)
50	100	SGL-MgDWD	0.735	(21.41, 2)	(0.14, 0.02)	1.661 (0.143)
		$L_{1}$-logistic	0.735	(20.13, 2)	(337.77, 22.07)	1.610 (0.110)
		OVR-SGL-gDWD	0.647	-	-	-
	1000	SGL-MgDWD	0.733	(21.25, 2)	(0, 0)	7.105 (0.205)
		$L_{1}$-logistic	0.566	(20.67, 2)	(3805.97, 265.82)	6.933 (0.205)
		OVR-SGL-gDWD	0.382	-	-	-
100	100	SGL-MgDWD	0.737	(21.82, 2)	(0.06, 0.01)	2.518 (0.099)
		$L_{1}$-logistic	0.721	(20, 2)	(173.17, 5.81)	2.418 (0.103)
		OVR-SGL-gDWD	0.609	-	-	-
	1000	SGL-MgDWD	0.737	(21.88, 2)	(5.4, 0.77)	12.371 (0.109)
		$L_{1}$-logistic	0.697	(20.15, 2)	(1859.51, 9.04)	12.279 (0.114)
		OVR-SGL-gDWD	0.214	-	-	-
200	100	SGL-MgDWD	0.738	(22, 2)	(0, 0)	5.191 (0.079)
		$L_{1}$-logistic	0.730	(20, 2)	(50.7, 0.08)	4.246 (0.100)
		OVR-SGL-gDWD	0.609	-	-	-
	1000	SGL-MgDWD	0.738	(21.98, 2)	(0.23, 0.04)	21.950 (0.241)
		$L_{1}$-logistic	0.730	(20, 2)	(523.08, 1.07)	22.158 (0.163)
		OVR-SGL-gDWD	0.490	-	-	-
400	100	SGL-MgDWD	0.740	(22, 2)	(0, 0)	7.025 (0.172)
		$L_{1}$-logistic	0.738	(20, 2)	(3.71, 3.48)	7.997 (0.122)
		OVR-SGL-gDWD	0.709	-	-	-
	1000	SGL-MgDWD	0.738	(22, 2)	(0.68, 0.11)	38.301 (0.200)
		$L_{1}$-logistic	0.734	(20, 2)	(38.84, 35.37)	41.059 (2.064)
		OVR-SGL-gDWD	0.556	-	-	-

**Table 3:** Signal for the coefficient estimates obtained from the SGL-MgDWD method with (q,τ)=(1,0.1) for the HIV data set. The symbols “+” and “-” denote positive and negative coefficient estimates, respectively, while “0” denotes a zero coefficient (i.e., an irrelevant variable).

	Non-HIV	HIVNBD	HIVBDS	HIVBDU
interception	+	+	-	+
miR-255b	-	+	-	+
miR-217	+	-	0	0
miR-25-star	0	+	+	-
miR-3136-5p	-	-	+	-
miR-3152-3p	+	-	-	+
miR-3159	-	-	-	+
miR-3171	0	+	-	-
miR-33b	-	-	-	+
miR-34c-3p	-	-	+	+
miR-3545-5p	-	+	-	+
miR-3654	-	-	-	+
miR-3924	0	-	+	-
miR-4307	0	-	+	0
miR-4474-5p	-	+	+	+
miR-4526	+	-	-	-
miR-4641	+	0	-	-
miR-4655-3p	+	0	-	-
miR-4680-5p	-	-	+	-
miR-4683	-	-	0	+
miR-589	-	+	+	-
miR-619	+	-	-	+
miR-660	+	0	-	+

## Appendix A. Proofs

### Appendix A.1. Proof of Lemma 1

**Proof.** For simplicity, we write $p_{j}=P\left(y=j|X\right)$ and $f_{k}=f_{k}\left(X\right)$. Using the Lagrange multiplier method, we define

$$\begin{array}{c} L\left(F\right)=E\left\{\sum_{k=1}^{K}𝟙\left\{y=k\right\}\phi_{q}\left\{F\left(X\right)\right\}|X=u\right\}+\mu 1_{K}^{⊤}F\left(X\right)=\sum_{k=1}^{K}p_{k}\phi_{q}\left(f_{k}\right)+\mu f_{k}. \end{array}$$

Then for each *k*,

(A1) $$\begin{array}{c} \frac{\partial L(F)}{\partial f_{k}}=\phi_{q}^{\prime }\left(f_{k}\right)p_{k}+\mu =0 \end{array}$$

with

$$\begin{array}{c} \phi_{q}^{\prime }\left(f_{j}\right)=\left\{\begin{array}{cc} -1, & f_{k}\leq Q\\ -{\left(Qf_{k}^{-1}\right)}^{q}, & f_{k}>Q. \end{array}\right. \end{array}$$

Without loss of generality, assume that $p_{1}>p_{2}\geq p_{3}\geq \cdots \geq p_{K-1}>p_{K}$. Note that $-1\leq \phi_{q}^{\prime }<0$, and so $p_{j}\geq -\phi_{q}^{\prime }\left(f_{k}\right)p_{k}=\mu >0$ and $\mu =p_{k}$ if and only if $f_{k}\leq Q$. If $\mu <p_{K}<p_{k}$, then $p_{K}\neq \mu$ when $f_{K}>Q$, which implies that $f_{k}>f_{K}>Q$ for all 1≤k≤K. Hence, substituting $\phi_{q}^{\prime }\left(f_{k}\right)=-{\left(Qf_{k}^{-1}\right)}^{q}$ into (A1) yields

$$\begin{array}{c} f_{k}=Q\sqrt[{q}]{p_{k}\mu^{-1}}>Q>0. \end{array}$$

However, $\sum_{k=1}^{K}f_{k}>0$, contradicting the sum-to-zero constraint. Therefore, $\mu =p_{K}<p_{k}$ for k<K and the result follows. □

### Appendix A.2. Proof of Theorem 1

**Lemma** **A1.**
*Under (C1), L(ϑ) exists, and it is convex on ϑ.*

**Proof.** The existence of L(ϑ) will be satisfied if

$$\begin{array}{c} E_{X|y}\left\{|\phi_{q}\left(Z^{⊤}\theta_{k}\right)||y=k\right\}=\int_{X}\left|\phi_{q}\left(Z^{⊤}\theta_{k}\right)\right|g_{k}\left(X\right)dX<\infty . \end{array}$$

We divide X into two disjoint subsets. Defining $X_{k}=\left\{X\in X|Z^{⊤}\theta_{k}>Q\right\}$, it is clear that

$$\begin{array}{c} \int_{X_{k}}\left|\phi_{q}\left(Z^{⊤}\theta_{k}\right)\right|g_{k}\left(X\right)dX\leq {\left(q+1\right)}^{-1}\int_{X_{k}}g_{k}\left(X\right)dX<\infty . \end{array}$$

Note that $0<\phi_{q}\left(u\right)<{\left(1+q\right)}^{-1}<1$ when u>Q. On the other hand, for $X_{k}^{c}=\left\{X\in X|Z^{⊤}\theta_{k}\leq Q\right\}$,

$$\begin{array}{c} \int_{X_{k}^{c}}|\phi_{q}\left(Z^{⊤}\theta_{k}\right)|g_{k}\left(X\right)dX\leq |1-a_{k}|+\sum_{j=1}^{p}b_{jk}\int_{X}\left|x_{j}\right|g_{k}\left(X\right)dX<\infty , \end{array}$$

if $E_{X|y}\left\{|x_{j}||y=k\right\}<\infty$ for all k∈Y. This completes the proof of the existence of L(ϑ). Recall that

$$\begin{array}{c} L\left(\vartheta \right)=\sum_{k=1}^{K}\pi_{k}\int_{X}\phi_{q}\left(Z^{⊤}\theta_{k}\right)g_{k}\left(X\right)dX, \end{array}$$

where $\phi_{q}\left(u\right)$ is a convex function of *u*, so its composition with the affine mapping $u=Z^{⊤}\theta_{k}$ is still convex in $\theta_{k}$. Clearly, $g_{k}\left(X\right),\;\pi_{k}>0$, so the non-negatively-weighted integral and sum both preserve convexity. □

**Lemma** **A2.**
*Existence of minimizers of L(ϑ) on $C=\left\{\vartheta \in R^{K(p+1)}|C\vartheta =0_{K}\right\}$, where $C=1_{K}^{⊤}⊗I_{p+1}$.*

**Proof.** By Jensen’s inequality, for any ϑ∈C, we have that

$$\begin{array}{c} L\left(\vartheta \right)\geq \phi_{q}\left(\sum_{k=1}^{K}\pi_{k}E\left\{Z^{⊤}\theta_{k}|y=k\right\}\right). \end{array}$$

Let $\mu =\mathrm{vec}\left\{{\left(\pi_{k}E\left\{z_{j}|y=k\right\}\right)}_{jk}\right\}$, where ${∥\mu ∥}_{2}\geq {\left(\sum_{k=1}^{K}\pi_{k}^{2}\right)}^{\frac{1}{2}}\geq K^{-\frac{1}{2}}>0$. For some C>0, we have that

$$\begin{array}{cc} L(\vartheta )\geq & \phi_{q}\left(\mu^{⊤}\vartheta \right)=𝟙\left\{\mu^{⊤}\vartheta <Q\right\}\left(1-\mu^{⊤}\vartheta \right)+𝟙\left\{\mu^{⊤}\vartheta \geq Q\right\}\varphi_{q}\left(\mu^{⊤}\vartheta \right)\\ \geq & 𝟙\left\{\mu^{⊤}\vartheta <Q\right\}|1-\left|\mu^{⊤}\vartheta \right||\\ = & 𝟙\left\{\mu^{⊤}\vartheta <-\left(C+1\right)\right\}\left(\right|\mu^{⊤}\vartheta |-1)+𝟙\left\{-\left(C+1\right)<\mu^{⊤}\vartheta <-1\right\}\left(\right|\mu^{⊤}\vartheta \left|-1\right)\\ \; & +𝟙\left\{-1<\mu^{⊤}\vartheta <Q\right\}\left(1-\right|\mu^{⊤}\vartheta \left|\right)\\ > & 𝟙\{∥\mu {∥}_{2}∥\vartheta {∥}_{2}>C+1\}C\\ = & 𝟙\left\{{∥\vartheta ∥}_{2}>\frac{C+1}{{∥\mu ∥}_{2}}\right\}C. \end{array}$$

Note that $1-\mu^{⊤}\vartheta >1-Q>0$ when $\mu^{⊤}\vartheta <Q$. By the Cauchy–Schwarz inequality, $-\mu^{⊤}\vartheta =|\mu^{⊤}{\vartheta |\leq ∥\mu ∥}_{2}{∥\vartheta ∥}_{2}$. Hence, if ${∥\vartheta ∥}_{2}>\frac{C+1}{{∥\mu ∥}_{2}}>0$, then L(ϑ)>C>0. The contrapositive of this result implies the existence of a minimizer in the unconstrained problem. That is, the closed set $\left\{\vartheta \in C|L(\vartheta )\leq C\right\}$ is bounded for some large enough *C*. This guarantees the existence of a solution, as desired. □

**Lemma** **A3.**
*Under (C1), S(ϑ) exists and*

$$\begin{array}{c} \frac{\partial L(\vartheta )}{\partial \vartheta }=S\left(\vartheta \right). \end{array}$$

**Proof.** The existence of S(ϑ) will follow if

$$\begin{array}{c} \int_{X}|\phi_{q}^{\prime }\left(Z^{⊤}\theta_{k}\right)z_{j}|\pi_{k}g_{k}\left(X\right)dX\leq \pi_{k}\int_{X}\left|z_{j}\right|g_{k}\left(X\right)dX<\infty \end{array}$$

for j=1,…,p+1. Note that $|\phi_{q}^{\prime }\left(u\right)|\leq 1$ when u>Q. For every $\theta_{kj}\in R$, $\phi_{q}\left(Z^{⊤}\theta_{k}\right)$ is a Lebesgue integrable function of X. For any u∈R, $\phi_{q}^{\prime }\left(u\right)$ exists and $|\phi_{q}^{\prime }\left(u\right)|\leq 1$. Hence, by the Leibniz integral rule, we have that

$$\begin{array}{cc} \frac{\partial }{\partial \theta_{jk}}\int_{X}\phi_{q}\left(Z^{⊤}\theta_{k}\right)\pi_{k}g_{k}\left(X\right)dX= & \int_{X}\frac{\partial \phi_{q}\left(Z^{⊤}\theta_{k}\right)}{\partial \theta_{jk}}\pi_{k}g_{k}\left(X\right)dX\\ = & \int_{X}\phi_{q}^{\prime }\left(Z^{⊤}\theta_{k}\right)z_{j}\pi_{k}g_{k}\left(X\right)dX \end{array}$$

and for any l≠k,

$$\begin{array}{c} \frac{\partial }{\partial \theta_{jl}}\int_{X}\phi_{q}\left(Z^{⊤}\theta_{k}\right)\pi_{k}g_{k}\left(X\right)dX=0, \end{array}$$

which is sufficient to show that

$$\begin{array}{c} \frac{\partial L(\vartheta )}{\partial \vartheta }=S\left(\vartheta \right). \end{array}$$

□

**Lemma** **A4.**
*Suppose (C1) is satisfied. Then (C2) implies that $b_{k}^{*}\neq 0$.*

**Proof.** We can rewrite $\phi_{q}\left(u\right)$ as

$$\begin{array}{cc} \phi_{q}\left(u\right)= & 𝟙\left\{u\leq Q\right\}\left(1-u\right)+𝟙\left\{u>Q\right\}\left(1-Q\right){\left(\frac{Q}{u}\right)}^{q}\\ = & \left\{-𝟙\left\{u\leq Q\right\}-𝟙\left\{u>Q\right\}{\left(\frac{Q}{u}\right)}^{q+1}\right\}u+𝟙\left\{u\leq Q\right\}+𝟙\left\{u>Q\right\}{\left(\frac{Q}{u}\right)}^{q}\\ = & \phi_{q}^{\prime }\left(u\right)u+𝟙\left\{u\leq Q\right\}+𝟙\left\{u>Q\right\}{\left(\frac{Q}{u}\right)}^{q}. \end{array}$$

Then for any $\gamma \in R^{p+1}$ and its corresponding $X_{k}=\left\{X\in X|Z^{⊤}\gamma >Q\right\}$, we have that

$$\begin{array}{cc} E\{𝟙 & \left\{y=k\right\}\phi_{q}\left(Z^{⊤}\gamma \right)\}\\ = & E\left\{𝟙\left\{y=k\right\}\phi_{q}^{\prime }\left(Z^{⊤}\gamma \right)Z^{⊤}\gamma \right\}+E\left\{𝟙\{y=k,\;Z^{⊤}\gamma \leq Q\}\right\}\\ \; & +E\left\{𝟙\left\{y=k,\;Z^{⊤}\gamma >Q\right\}{\left(\frac{Q}{Z^{⊤}\gamma }\right)}^{q}\right\}\\ = & S_{k}^{⊤}\left(\gamma \right)\gamma +\mathrm{Pr}\left\{y=k,\;X\notin X_{k}\right\}+E\left\{𝟙\left\{y=k,\;X\in X_{k}\right\}{\left(\frac{Q}{Z^{⊤}\gamma }\right)}^{q}\right\}\\ = & S_{k}^{⊤}\left(\gamma \right)\gamma +\pi_{k}\left(1-E\left\{𝟙\left\{X\in X_{k}\right\}\left\{1-{\left(\frac{Q}{Z^{⊤}\gamma }\right)}^{q}\right\}|y=k\right\}\right). \end{array}$$

Let $\vartheta^{*}\in C$ be a local minimizer. It follows that $PS(\vartheta^{*})=0$ and $\sum_{k=1}^{K}S_{k}^{⊤}\left(\theta_{k}^{*}\right)\theta_{k}^{*}=S^{⊤}\left(\vartheta^{*}\right)\vartheta^{*}=0$ since $\vartheta^{*}=P\vartheta^{*}$ and $P=\left(I_{K}-K^{-1}1_{K}1_{K}^{⊤}\right)⊗I_{p+1}$. Therefore,

(A2) $$\begin{array}{cc} L(\vartheta^{*})= & E\left\{𝟙\left\{y=k\right\}\phi_{q}\left(Z^{⊤}\theta_{k}^{*}\right)\right\}\\ = & \sum_{k=1}^{K}\pi_{k}\left(1-E\left\{𝟙\left\{X\in X_{k}^{*}\right\}\left\{1-{\left(\frac{Q}{Z^{⊤}\theta_{k}^{*}}\right)}^{q}\right\}|y=k\right\}\right)\\ = & \sum_{k=1}^{K}\pi_{k}\left(1-\mathrm{Pr}\left\{X\in X_{k}^{*}|y=k\right\}E\left\{1-{\left(\frac{Q}{Z^{⊤}\theta_{k}^{*}}\right)}^{q}|y=k,\;X\in X_{k}^{*}\right\}\right). \end{array}$$

For any $\gamma \in R^{p+1}$ and its corresponding $X_{k}=\left\{X\in X|Z^{⊤}\gamma >Q\right\}$, we always have that

$$\begin{array}{c} 0<E\left\{{\left(\frac{Q}{Z^{⊤}\gamma }\right)}^{q}|y=k,\;X\in X_{k}\right\}<1. \end{array}$$

If $\gamma =0_{p+1}$, then $X_{k}=⌀$ so that $\mathrm{Pr}\left\{y=k,\;X\notin X_{k}\right\}=\pi_{k}$ and $\mathrm{Pr}\{y=k,\;X\in X_{k}\}=0$. If $\gamma_{1}\leq Q$ and $\gamma_{/1}=0_{p}$, then $X_{k}=⌀$, giving the same conclusions as the previous case. If $\gamma_{1}>Q$ and $\gamma_{/1}=0_{p}$, then $X_{k}=X$ so that $\mathrm{Pr}\{y=k,\;X\notin X_{k}\}=0$ and $\mathrm{Pr}\left\{y=k,\;X\in X_{k}\right\}=\pi_{k}$. Consequently, when $0<\mathrm{Pr}\{X\in X_{k}|y=k\}<1$, then neither $X_{k}$ nor X equal ⌀, so $b_{k}\neq 0$ follows. Note that $\mathrm{Pr}\{X\notin X_{k}|y=k\}>0$ implies that $\mathrm{Pr}\{0<Z^{⊤}\gamma \leq Q|y=k\}>0$ or $\mathrm{Pr}\{Z^{⊤}\gamma \leq 0|y=k\}>0$, and so special attention should be paid to bounded random variables. □

**Lemma** **A5.**
*Under (C1), H(ϑ) exists and*

$$\begin{array}{c} \frac{\partial^{2}L\left(\vartheta \right)}{\partial \vartheta \partial \vartheta^{⊤}}=H\left(\vartheta \right). \end{array}$$

*Furthermore, $H\left(\vartheta^{*}\right)≻O_{K(p+1)}$ when (C2) and (C3) hold.*

**Proof.** The existence of H(ϑ) follows if its all entries are absolutely integrable, that is, for any j,k=1,…,p+1,

$$\begin{array}{c} \int_{X}|𝟙\{Z^{⊤}\theta_{k}>Q\}\varphi_{q}^{\prime \prime }\left(Z^{⊤}\theta_{k}\right)z_{j}z_{l}|\pi_{k}g_{k}\left(X\right)dX\\ \;\leq \left(q+q^{-1}+2\right)\int_{X_{k}^{c}}\left|z_{j}z_{l}\right|g_{k}\left(X\right)dX\\ \;<\infty . \end{array}$$

Equivalently, the result follows if $E_{X|y}\left\{|z_{j}z_{l}||y=k\right\}<\infty$ for all k∈Y. Note that $0<\varphi_{q}^{\prime \prime }\left(u\right)\leq q+q^{-1}+2$ when u>Q. Let η be a test function belonging to the Schwartz space D. Then $\eta^{\prime }\in D$ with some support denoted by $\mathrm{supp}(\eta^{\prime })$. Clearly, $\phi_{q}^{\prime }\left(u\right)$ is not differentiable at *Q* but is Lipschitz continuous. Therefore, the measurable function $S_{k}\left(\theta_{k}\right)$ is a locally integrable function of $\theta_{k}$. Then the (regular) generalized functions $S_{k}\left(\theta_{k}\right)$ belong to the dual space of D. For the distributional derivative of $S_{k}\left(\theta_{k}\right)$ with respect to $\theta_{jk}$, we have that

$$\begin{array}{cc} \left|\langle \frac{\partial S_{k}\left(\theta_{k}\right)}{\partial \theta_{jk}},\eta \left(\theta_{jk}\right)\rangle \right|= & \left|-\langle S_{k}\left(\theta_{k}\right),\frac{d\eta (\theta_{jk})}{d\theta_{jk}}\rangle \right|\\ \leq & \int_{R}\left|S_{k}\left(\theta_{k}\right)\eta^{\prime }\left(\theta_{jk}\right)\right|d\theta_{jk}\\ \leq & \max_{\theta_{jk}\in \mathrm{supp}\left(\eta^{\prime }\right)}|\eta^{\prime }\left(\theta_{jk}\right)|\int_{\mathrm{supp}(\eta^{\prime })}\left|S_{k}\left(\theta_{k}\right)\right|d\theta_{jk}\\ < & \infty \end{array}$$

implying that the function $f\left(\theta_{jk},X\right)=\phi_{q}^{\prime }\left(Z^{⊤}\theta_{k}\right)Z\pi_{k}g_{k}\left(X\right)\eta^{\prime }\left(\theta_{jk}\right)$ is integrable on R×X. Therefore, by Fubini’s Theorem,

$$\begin{array}{cc} \langle \frac{\partial S_{k}\left(\theta_{k}\right)}{\partial \theta_{jk}},\eta \left(\theta_{jk}\right)\rangle = & -\langle S_{k}\left(\theta_{k}\right),\frac{d\eta (\theta_{jk})}{d\theta_{jk}}\rangle \\ = & \int_{X}-\langle \phi_{q}^{\prime }\left(Z^{⊤}\theta_{k}\right)Z\pi_{k}g_{k}\left(X\right),\frac{d\eta (\theta jk)}{d\theta_{jk}}\rangle dX\\ = & \int_{X}\langle \frac{\partial \phi_{q}^{\prime }\left(Z^{⊤}\theta_{k}\right)}{\partial \theta_{jk}}Z\pi_{k}g_{k}\left(X\right),\eta \left(\theta_{jk}\right)\rangle dX\\ = & \langle E\left\{\frac{\partial \phi_{q}^{\prime }\left(Z^{⊤}\theta_{k}\right)}{\partial \theta_{jk}}Z𝟙\left\{y=k\right\}\right\},\eta \left(\theta_{jk}\right)\rangle , \end{array}$$

which implies that

$$\begin{array}{c} \frac{\partial S_{k}\left(\theta_{k}\right)}{\partial \theta_{jk}}=E\left\{\frac{\partial \phi_{q}^{\prime }\left(Z^{⊤}\theta_{k}\right)}{\partial \theta_{jk}}Z𝟙\left\{y=k\right\}\right\}. \end{array}$$

Recall that $\phi_{q}^{\prime }$ can be written as

$$\begin{array}{c} \phi_{q}^{\prime }\left(u\right)=\varphi_{q}^{\prime }\left(u\right)𝟙\left\{u>Q\right\}+\left(-1\right)𝟙\left\{u\leq Q\right\}=\left(\varphi_{q}^{\prime }\left(u\right)+1\right)𝟙\left\{u>Q\right\}-1, \end{array}$$

which contains a Schwartz product between the differentiable function $\varphi_{q}^{\prime }\left(u\right)$ and the generalized function 𝟙{u>Q}. Note that

$$\begin{array}{cc} 𝟙\{Z^{⊤}\theta_{k}>Q\}= & 𝟙\left\{z_{j}>0,\;\theta_{jk}>c_{jk}\right\}+𝟙\left\{z_{j}\leq 0,\;\theta_{jk}\leq c_{jk}\right\}\\ = & \left(2𝟙\left\{z_{j}>0\right\}-1\right)𝟙\left\{\theta_{jk}>c_{jk}\right\}+\left(1-𝟙\left\{z_{j}>0\right\}\right)\\ = & \mathrm{sign}\left(z_{j}\right)𝟙\left\{\theta_{jk}>c_{jk}\right\}+𝟙\left\{z_{j}\leq 0\right\}, \end{array}$$

where $c_{jk}=\left(Q-\sum_{l\neq j}z_{l}\theta_{lk}\right)/z_{j}$ and

$$\begin{array}{cc} \frac{\partial 𝟙\{Z^{⊤}\theta_{k}>Q\}}{\partial \theta_{jk}}+0 & =\mathrm{sign}\left(z_{j}\right)\delta \left(\theta_{jk}-c_{jk}\right)\\ & =\mathrm{sign}\left(z_{j}\right)\left|z_{j}\right|\delta \left(Z^{⊤}\theta_{k}-Q\right)\\ & =z_{j}\delta \left(Z^{⊤}\theta_{k}-Q\right), \end{array}$$

where δ(x) is the Dirac delta function and the distributional derivative of 𝟙{x>0}. Recall that δ(cx)=δ(x)/|c| and f(x)δ(x−c)=f(c)δ(x−c) for some constant *c* and function *f*. Thus, by the product rule for the distributional derivative of the Schwartz product,

$$\begin{array}{cc} \frac{\partial \phi_{q}^{\prime }\left(Z^{⊤}\theta_{k}\right)}{\partial \theta_{jk}}= & \frac{\partial (\varphi_{q}^{\prime }\left(Z^{⊤}\theta_{k}\right)+1)}{\partial \theta_{jk}}𝟙\left\{Z^{⊤}\theta_{k}>Q\right\}+\left(\varphi_{q}^{\prime }\left(Z^{⊤}\theta_{k}\right)+1\right)\frac{\partial 𝟙\{Z^{⊤}\theta_{k}>Q\}}{\partial \theta_{jk}}\\ = & \varphi_{q}^{\prime \prime }\left(Z^{⊤}\theta_{k}\right)z_{j}𝟙\left\{Z^{⊤}\theta_{k}>Q\right\}+\left(\varphi_{q}^{\prime }\left(Z^{⊤}\theta_{k}\right)+1\right)z_{j}\delta \left(Z^{⊤}\theta_{k}-Q\right)\\ = & \varphi_{q}^{\prime \prime }\left(Z^{⊤}\theta_{k}\right)z_{j}𝟙\left\{Z^{⊤}\theta_{k}>Q\right\}. \end{array}$$

Substituting the above expression, we obtain

$$\begin{array}{c} \frac{\partial S_{k}\left(\theta_{k}\right)}{\partial \theta_{jk}}=E\left\{\varphi_{q}^{\prime \prime }\left(Z^{⊤}\theta_{k}\right)Zz_{j}𝟙\left\{Z^{⊤}\theta_{k}>Q\right\}𝟙\left\{y=k\right\}\right\}. \end{array}$$

Similarly, for l≠k, we have the distributional derivative

$$\begin{array}{c} \frac{\partial S_{k}\left(\theta_{k}\right)}{\partial \theta_{jl}}=0. \end{array}$$

Recall that the distributional derivative does not depend on the order of differentiation and agrees with the classical derivative whenever the latter exists. To summarize, we have that

$$\begin{array}{cc} H_{k}\left(\theta_{k}\right)= & \frac{\partial^{2}L\left(\vartheta \right)}{\partial \theta_{k}\partial \theta_{k}^{⊤}}=\frac{\partial S_{k}\left(\theta_{k}\right)}{\partial \theta_{k}^{⊤}},\;H\left(\vartheta \right)=\overset{K}{\underset{k=1}{⨁}}H_{k}\left(\theta_{k}\right). \end{array}$$

The $H_{k}\left(\theta_{k}\right)$ are symmetric matrices, so H(ϑ) is also symmetric. In the sense of generalized functions, differentiation is a continuous operation with respect to convergence in $D^{\prime }$. Therefore, $\phi_{0}^{\prime }=\lim_{q\to 0}\phi_{q}^{\prime }=-𝟙\left\{u\leq 0\right\}$ and $\phi_{0}^{\prime \prime }=\lim_{q\to 0}\phi_{q}^{\prime \prime }=\delta \left(u\right)$; $\phi_{\infty }^{\prime }=\lim_{q\to \infty }\phi_{q}^{\prime }=-𝟙\left\{u\leq 1\right\}$ and $\phi_{\infty }^{\prime \prime }=\lim_{q\to \infty }\phi_{q}^{\prime \prime }=\delta \left(u-1\right)$, which coincides with results from the hinge loss. Next, $H\left(\vartheta \right)≻O_{K(p+1)}$ if and only if both $H_{1}\left(\theta_{1}\right)$ and its Schur complement ${⨁}_{k=2}^{K}H_{k}\left(\theta_{k}\right)$ are both symmetric and positive definite. We can deduce that $H\left(\vartheta \right)≻O_{K(p+1)}$ if and only if $H_{k}\left(\theta_{k}\right)≻O_{p+1}$ for all *k*. Note that there exists c>0 such that $\varphi_{q}^{\prime \prime }\left(Z^{⊤}\theta_{k}\right)\geq c$ on $X_{k}$. Then for any $\gamma \in R^{p+1}$,

$$\begin{array}{cc} \gamma^{⊤}H_{k}\left(\theta_{k}\right)\gamma = & \pi_{k}\int_{X_{k}}\varphi_{q}^{\prime \prime }\left(Z^{⊤}\theta_{k}\right){\left(Z^{⊤}\gamma \right)}^{2}g_{k}\left(X\right)dX\\ \geq & c\mathrm{Pr}\left\{X\in X_{k},\;y=k\right\}E\left\{{\left(Z^{⊤}\gamma \right)}^{2}|X\in X_{k},\;y=k\right\}\\ \geq & c\mathrm{Pr}\left\{X\in X_{k},\;y=k\right\}\left(\gamma_{0}^{2}+\gamma_{1}^{⊤}\mathrm{Var}\left\{X|X\in X_{k},\;y=k\right\}\gamma_{1}\right), \end{array}$$

which implies that $\gamma^{⊤}H_{k}\left(\theta_{k}\right)\gamma =0$ if and only if $\gamma =0_{p+1}$ when $\mathrm{Var}\{X|X\in X_{k},\;y=k\}$ is assumed to be non-singular. Assuming that Var{X|y=k}≻O implies that $\mathrm{Var}\{X|X\in X_{k},\;y=k\}⪰O$. □

**Proof** **of** **Theorem** **1** By Lemma A2, a minimizer $\vartheta^{*}\in C$ exists with $b_{k}^{*}\neq 0_{p}$ (by Lemma A4) and $H\left(\vartheta^{*}\right)≻O_{K(p+1)}$ (by Lemma A5). By the second-order Lagrange condition and the convexity of L(ϑ) (by Lemma A1), a minimizer of the population MgDWD loss is unique. Recall from (A2) that

$$\begin{array}{cc} L(\vartheta^{*})= & E\left\{𝟙\left\{y=k\right\}\phi_{q}\left(Z^{⊤}\theta_{k}^{*}\right)\right\}\\ = & \sum_{k=1}^{K}\pi_{k}\left(1-E\left\{𝟙\left\{X\in X_{k}^{*}\right\}\left\{1-{\left(\frac{Q}{Z^{⊤}\theta_{k}^{*}}\right)}^{q}\right\}|y=k\right\}\right)\\ = & \sum_{k=1}^{K}A\left(k,q\right)\pi_{k}. \end{array}$$

It follows that

$$\begin{array}{cc} 0\leq & E\left\{𝟙\left\{X\in X_{k}^{*}\right\}\left\{1-{\left(\frac{Q}{Z^{⊤}\theta_{k}^{*}}\right)}^{q}\right\}|y=k\right\}\\ < & E\left\{𝟙\left\{Z^{⊤}\gamma >1+q^{-1}\right\}+𝟙\left\{Q<Z^{⊤}\gamma \leq 1+q^{-1}\right\}\left\{1-{\left(\frac{Q}{1+q^{-1}}\right)}^{q}\right\}|y=m\right\}\\ = & \mathrm{Pr}\left\{Z^{⊤}\gamma >Q|y=m\right\}-\mathrm{Pr}\left\{Q<Z^{⊤}\gamma \leq Q^{-1}|y=m\right\}Q^{2q}\\ \leq & 1 \end{array}$$

and

$$\begin{array}{cc} 1\geq & E\left\{𝟙\left\{X\in X_{k}^{*}\right\}\left\{1-{\left(\frac{Q}{Z^{⊤}\theta_{k}^{*}}\right)}^{q}\right\}|y=k\right\}\\ > & E\left\{𝟙\left\{Z^{⊤}\theta_{k}^{*}>1+\epsilon \right\}\left\{1-{\left(\frac{Q}{1+\epsilon }\right)}^{q}\right\}|y=k\right\}\\ \geq & \underset{\epsilon >0}{\sup}\left\{1-{\left(\frac{Q}{1+\epsilon }\right)}^{q}\right\}\mathrm{Pr}\left\{Z^{⊤}\theta_{k}^{*}>1+\epsilon |y=m\right\}\\ \geq & 0. \end{array}$$

Consequently, 0≤u(k,q)≤A(k,q)≤v(k,q)≤1. Note that $\lim_{q\to \infty }{\left(1+\epsilon \right)}^{-q}Q^{q}=e^{-1}$ when ϵ=0 and $\lim_{q\to \infty }{\left(1+\epsilon \right)}^{-q}Q^{q}=0$ when ϵ>0. The difference between these two results is attributed to pointwise convergence. Let $f_{m}=1-A\left(k,m\right)\in D^{\prime }$ with m=1,2,… and η∈D. By Fubini’s theorem and the dominated convergence theorem,

$$\begin{array}{cc} \lim_{m\to \infty }\langle f_{m},\eta \rangle = & \lim_{m\to \infty }\langle E\left\{𝟙\left\{X\in X_{k}^{*}\right\}{\left(\frac{Q}{Z^{⊤}\theta_{k}^{*}}\right)}^{q}|y=k\right\},\eta \left(\gamma \right)\rangle \\ = & \lim_{m\to \infty }E\left\{\langle 𝟙\left\{X\in X_{k}^{*}\right\}{\left(\frac{Q}{Z^{⊤}\theta_{k}^{*}}\right)}^{q},\eta \left(\theta_{k}^{*}\right)\rangle |y=k\right\}\\ = & E\left\{\lim_{m\to \infty }\langle 𝟙\left\{X\in X_{k}^{*}\right\}{\left(\frac{Q}{Z^{⊤}\theta_{k}^{*}}\right)}^{q},\eta \left(\theta_{k}^{*}\right)\rangle |y=k\right\}\\ = & 0=\langle 0,\eta (\theta_{k}^{*})\rangle . \end{array}$$

Similarly,

$$\begin{array}{cc} \lim_{m\to 0}\langle f_{m},\eta \rangle = & E\left\{\lim_{m\to 0}\langle 𝟙\left\{X\in X_{k}^{*}\right\}{\left(\frac{Q}{Z^{⊤}\theta_{k}^{*}}\right)}^{q},\eta \left(\gamma \right)\rangle |y=k\right\}\\ = & E\left\{\langle 𝟙\left\{Z^{⊤}\theta_{k}^{*}>0\right\},\eta \left(\gamma \right)\rangle |y=k\right\}\\ = & \langle E\left\{𝟙\{Z^{⊤}\theta_{k}^{*}>0\}|y=k\right\},\eta \left(\theta_{k}^{*}\right)\rangle \\ = & \langle \mathrm{Pr}\left\{Z^{⊤}\theta_{k}^{*}>0|y=k\right\},\eta \left(\theta_{k}^{*}\right)\rangle , \end{array}$$

hence

$$\begin{array}{c} A\left(k,\infty \right)=\lim_{q\to \infty }A\left(k,q\right)=\mathrm{Pr}\left\{X\notin X_{k}^{*}|y=k\right\},\;\mathrm{and}\;A\left(k,0\right)=\lim_{q\to 0}A\left(k,q\right)=1. \end{array}$$

As a result, A(k,∞) coincides with the population hinge/SVM loss and A(k,0) is independent of $\theta_{k}^{*}$. □

### Appendix A.3. Proof of Lemma 2

**Proof.** By the definition of $\tilde{P}$,

$$\begin{array}{c} \tilde{P}\left\{PS(\vartheta^{*})\right\}=\tau {∥PS\left(\vartheta^{*}\right)∥}_{\infty }+\left(1-\tau \right)\max_{j}\left\{{∥P_{K}S\left(\alpha^{*}\right)∥}_{2},{∥P_{K}S\left(\beta_{j}^{*}\right)∥}_{2}\right\}, \end{array}$$

where

$$\begin{array}{c} P_{K}S\left(\alpha^{*}\right)=P_{K}{\left(E\circ \phi_{q}^{\prime }\left\{F\left(\vartheta^{*}\right)\right\}\right)}^{⊤}1_{K}=\frac{1}{N}\sum_{i=1}^{N}P_{K}\mathrm{diag}\left\{E_{i}\right\}\phi_{q}^{\prime }\left(F_{i}^{*}\right),\\ P_{K}S\left(\beta_{j}^{*}\right)=P_{K}{\left(E\circ \phi_{q}^{\prime }\left\{F\left(\vartheta^{*}\right)\right\}\right)}^{⊤}x_{j}=\frac{1}{N}\sum_{i=1}^{N}x_{ij}P_{K}\mathrm{diag}\left\{E_{i}\right\}\phi_{q}^{\prime }\left(F_{i}^{*}\right), \end{array}$$

$P_{K}=\left(p_{1},\ldots ,p_{K}\right)$ with $p_{k}=\left(p_{lk}\right)=𝟙\left\{l=k\right\}-K^{-1}$, and

$$\begin{array}{c} E\left\{P_{K}S\left(\alpha^{*}\right)\right\}=P_{K}S\left(\alpha^{*}\right)=0_{K},\;E\left\{P_{K}S\left(\beta_{j}^{*}\right)\right\}=P_{K}S\left(\beta_{j}^{*}\right)=0_{K}. \end{array}$$

Denoting

$$\begin{array}{c} d_{ik}=\left\{p_{k}^{⊤}\mathrm{diag}\left\{E_{i}\right\}\phi_{q}^{\prime }\left(F_{i}^{*}\right)\right\}=\sum_{l=1}^{K}\left(𝟙\left\{y_{i}=k\right\}-\frac{1}{K}\right)e_{il}\phi_{q}^{\prime }\left(f_{il}^{*}\right), \end{array}$$

we have that $|d_{ik}|\leq 1-K^{-1}$. Note that the $d_{ik}$ are *N* i.i.d. random variables with

$$\begin{array}{c} \frac{1}{N}\sum_{i=1}^{N}E\left(d_{ik}\right)=p_{k}^{⊤}S\left(\alpha^{*}\right)=0\;\mathrm{and}\;\frac{1}{N}\sum_{i=1}^{N}E\left(d_{ik}x_{ij}\right)=p_{k}^{⊤}S\left(\beta_{j}^{*}\right)=0. \end{array}$$

By Hoeffding’s inequality, we have that

(A3) $$\begin{array}{c} \mathrm{Pr}\left\{|p_{k}^{⊤}S\left(\alpha^{*}\right)|>c_{1}\left(1-\frac{1}{K}\right)\sqrt{\frac{2\log(pK)}{N}}\right\}\leq 2{\left(pK\right)}^{-c_{1}^{2}}, \end{array}$$

where $c_{1}>1$. Regarding the $d_{ik}x_{ij}$, we have that

$$\begin{array}{c} E\exp\left\{d_{ik}x_{ij}\right\}\leq E\exp\{\left(1-K^{-1}\right)|x_{ij}\left|\right\}\leq \exp\{4{\left(1-K^{-1}\right)}^{2}\varsigma_{1}^{2}\kappa^{2}\}, \end{array}$$

which implies that the $d_{ik}x_{ij}$ are *N* independent sub-Gaussian random variables with variance proxy ${\left(1-K^{-1}\right)}^{2}\varsigma_{1}^{2}\kappa^{2}$. Taking $c_{1}>1$, we have that

(A4) $$\begin{array}{c} \mathrm{Pr}\left\{|p_{k}^{⊤}S\left(\beta_{j}^{*}\right)|>c_{1}\varsigma_{1}\kappa \left(1-\frac{1}{K}\right)\sqrt{\frac{2\log(pK)}{N}}\right\}\leq 2{\left(pK\right)}^{-c_{1}^{2}}. \end{array}$$

Then by (A3) and (A4),

(A5) $$\begin{array}{c} \mathrm{Pr}\left\{\max_{j}\left\{|p_{k}^{⊤}S\left(\alpha^{*}\right)\left|,\right|p_{k}^{⊤}S\left(\beta_{j}^{*}\right)|\right\}>\Lambda_{1}\right\}\leq 2{\left(pK\right)}^{-c_{1}^{2}} \end{array}$$

with

$$\begin{array}{c} \Lambda_{1}=\max\left\{\varsigma_{1}\kappa ,1\right\}c_{1}\left(1-\frac{1}{K}\right)\sqrt{\frac{2\log(pK)}{N}}. \end{array}$$

Taking a union bound over the Kp entries of $PS(\beta^{*})$ yields that

$$\begin{array}{cc} \mathrm{Pr}\left\{{∥PS\left(\vartheta^{*}\right)∥}_{\infty }\geq \Lambda_{1}\right\}= & \mathrm{Pr}\left\{\max_{j,k}\left\{|\frac{1}{N}\sum_{i=1}^{N}p_{k}^{⊤}S\left(\alpha^{*}\right)|,|\frac{1}{N}\sum_{i=1}^{N}p_{k}^{⊤}S\left(\beta_{j}^{*}\right)|\right\}\geq \Lambda_{1}\right\}\\ \leq & 2K\left(p+1\right){\left(Kp\right)}^{-c_{1}^{2}}. \end{array}$$

On one hand,

$$\begin{array}{c} ∥P\mathrm{diag}\left\{E_{i}\right\}\phi_{q}^{\prime }\left(F_{i}^{*}\right){∥}_{2}^{2}=∥\left(E_{i}-K^{-1}\right)\circ \phi_{q}^{\prime }\left(F_{i}^{*}\right){∥}_{2}^{2}\leq \sum_{l=1}^{K}{\left(e_{il}-K^{-1}\right)}^{2}\cdot 1=1-K^{-1}, \end{array}$$

so for any $\gamma \in R^{K}$,

$$\begin{array}{c} |\gamma^{⊤}P\mathrm{diag}\left\{E_{i}\right\}\phi_{q}^{\prime }\left(F_{i}^{*}\right){|\leq ∥\gamma ∥}_{2}\sqrt{1-\frac{1}{K}} \end{array}$$

and $E\{\gamma^{⊤}P\mathrm{diag}\left\{E_{i}\right\}\phi_{q}^{\prime }\left(F_{i}^{*}\right)\}=0$. Applying Hoeffding’s lemma,

$$\begin{array}{c} E\exp\left\{\gamma^{⊤}P_{K}S\left(\alpha^{*}\right)\right\}=\prod_{i=1}^{N}E\exp\left\{\frac{1}{N}\gamma^{⊤}P_{K}\mathrm{diag}\left\{E_{i}\right\}\phi_{q}^{\prime }\left(F_{i}^{*}\right)\right\}\leq \exp\left\{\frac{{∥\gamma ∥}_{2}^{2}}{2N}\left(1-\frac{1}{K}\right)\right\}. \end{array}$$

Applying a square root to Theorem 2.1 of [31] with $c_{2}>1$, we have that

(A6) $$\begin{array}{c} \mathrm{Pr}\left\{∥PS\left(\alpha^{*}\right){∥}_{2}\geq \sqrt{\frac{K-1}{N}}+c_{2}\sqrt{\left(1-\frac{1}{K}\right)\frac{2\log(p)}{N}}\right\}\leq p^{-c_{2}^{2}}. \end{array}$$

On the other hand, since the $x_{ij}$ are *N* independent sub-Gaussian random variables with variance proxy $\varsigma_{1}^{2}\kappa^{2}$,

$$\begin{array}{cc} E\exp\{\gamma^{⊤}PS\left(\beta_{j}^{*}\right)\}= & \prod_{i=1}^{N}E\exp\left\{\frac{x_{ij}}{N}\left\{\gamma^{⊤}P\mathrm{diag}\left\{E_{i}\right\}\phi_{q}^{\prime }\left(F_{i}^{*}\right)\right\}\right\}\\ \leq & \prod_{i=1}^{N}E\exp\left\{\sqrt{1-\frac{1}{K}}\frac{{∥\gamma ∥}_{2}}{N}\left|x_{ij}\right|\right\}\\ \leq & =\exp\left\{\frac{{∥\gamma ∥}_{2}^{2}}{2}\left(1-\frac{1}{K}\right)\frac{8\varsigma_{1}^{2}\kappa^{2}}{N}\right\} \end{array}$$

and $E\left\{P_{K}S\left(\beta_{j}^{*}\right)\right\}=0_{K}$. Similarly, we have that

(A7) $$\begin{array}{c} \mathrm{Pr}\left\{∥PS\left(\beta_{j}^{*}\right){∥}_{2}\geq 2\sqrt{2}\varsigma_{1}\kappa \left\{\sqrt{\frac{K-1}{N}}+c_{2}\sqrt{\left(1-\frac{1}{K}\right)\frac{2\log(p)}{N}}\right\}\right\}\leq p^{-c_{2}^{2}} \end{array}$$

for a constant $c_{2}>1$. Therefore, by (A6) and (A7),

$$\begin{array}{c} \mathrm{Pr}\left\{\max_{j}\left\{∥PS\left(\alpha^{*}\right){∥}_{2},{∥PS\left(\beta_{j}^{*}\right)∥}_{2}\right\}\geq \Lambda_{2}\right\}\leq p^{-c_{2}^{2}} \end{array}$$

with

$$\begin{array}{c} \Lambda_{2}=\max\left\{2\sqrt{2}\varsigma_{1}\kappa ,1\right\}\left\{\sqrt{\frac{K-1}{N}}+c_{2}\sqrt{\left(1-\frac{1}{K}\right)\frac{2\log(p)}{N}}\right\}. \end{array}$$

Applying the union bound to (A5), it follows that

$$\begin{array}{c} \mathrm{Pr}\left\{\tilde{P}\left\{PS(\vartheta^{*})\right\}\geq \tau \Lambda_{1}+\left(1-\tau \right)\Lambda_{2}\right\}\leq 2K\left(p+1\right){\left(pK\right)}^{1-c_{1}^{2}}+p^{1-c_{2}^{2}}, \end{array}$$

and the desired result follows. □

### Appendix A.4. Proof of Theorem 2

**Lemma** **A6.**
*Suppose that $\lambda =c_{0}\sqrt{\frac{\log(pK)}{N}}$. Then $\hat{\vartheta }-\vartheta^{*}\in U$, where*

$$\begin{array}{c} U=\left\{\delta \in R^{K(p+1)}|\frac{\tau }{1-\tau }{∥\delta_{E_{+}}∥}_{1}+\sum_{j\in G_{+}}{∥\delta_{j}∥}_{2}\geq C_{0}\left(\frac{\tau }{1-\tau }{∥\delta_{E^{c}}∥}_{1}+\sum_{j\notin G}{∥\delta_{j}∥}_{2}\right)\right\}, \end{array}$$

*$C_{0}=\frac{\left(c_{0}-1\right)}{\left(c_{0}+1\right)}$, $E^{c}$ denotes the complement of E, $E_{+}=E\cup \left\{l=1+\left(k-1\right)\left(p+1\right)|k=1,\ldots ,K\right\}$, and $G_{+}=G\cup \left\{0\right\}$.*

**Proof.** Since $\hat{\vartheta }=\vartheta^{*}+\delta$ is the minimizer, we have that

(A8) $$\begin{array}{cc} L\left(\vartheta^{*}\right)+\lambda P\left(\beta^{*}\right)\geq & L\left(\hat{\vartheta }\right)+\lambda P\left(\hat{\beta }\right)\\ \lambda \left\{P\left(\beta^{*}\right)-P\left(\beta^{*}+\tilde{\delta }\right)\right\}\geq & L\left(\vartheta^{*}+\delta \right)-L\left(\vartheta^{*}\right), \end{array}$$

where $\beta^{*}$ is the vector $\vartheta^{*}$ without the $a_{k}$ components, replacing $\tilde{\delta }$ for δ. Then

$$\begin{array}{cc} P\left(\beta^{*}\right)-P\left(\beta^{*}+\tilde{\delta }\right)= & \tau \left({∥\beta_{E}^{*}∥}_{1}-{∥\beta_{E}^{*}+{\tilde{\delta }}_{E}∥}_{1}-{∥{\tilde{\delta }}_{E^{c}}∥}_{1}\right)\\ \; & +\left(1-\tau \right)\left(\sum_{j\in G}{∥\beta_{j}^{*}∥}_{2}-\sum_{j\in G}{∥\beta_{j}^{*}+\delta_{j}∥}_{2}-\sum_{j\notin G}{∥\delta_{j}^{*}∥}_{2}\right)\\ \leq & \tau \left({∥{\tilde{\delta }}_{E}∥}_{1}-{∥{\tilde{\delta }}_{E^{c}}∥}_{1}\right)+\left(1-\tau \right)\left(\sum_{j\in G}{∥\delta_{j}∥}_{2}-\sum_{j\notin G}{∥\delta_{j}^{*}∥}_{2}\right)\\ \leq & \tau \left({∥\delta_{E_{+}}∥}_{1}-{∥\delta_{E^{c}}∥}_{1}\right)+\left(1-\tau \right)\left(\sum_{j\in G_{+}}{∥\delta_{j}∥}_{2}-\sum_{j\notin G}{∥\delta_{j}∥}_{2}\right). \end{array}$$

By the convexity of *L*,

$$\begin{array}{c} L\left(\vartheta^{*}+\delta \right)-L\left(\vartheta^{*}\right)\geq \langle S\left(\vartheta^{*}\right),\delta \rangle \geq -\bar{P}\left\{PS\left(\vartheta^{*}\right)\right\}P\left(\delta \right)\geq -\frac{\lambda }{c_{0}}P\left(\delta \right). \end{array}$$

Note that

$$\begin{array}{c} P\left(\delta \right)=\tau \left({∥\delta_{E_{+}}∥}_{1}+{∥\delta_{E^{c}}∥}_{1}\right)+\left(1-\tau \right)\left(\sum_{j\in G_{+}}{∥\delta_{j}∥}_{2}+\sum_{j\notin G}{∥\delta_{j}∥}_{2}\right). \end{array}$$

Combining the above results, we have that

$$\begin{array}{c} \lambda \left\{P\left(\vartheta^{*}\right)-P\left(\vartheta^{*}+\delta \right)\right\}\geq \left\{L\left(\vartheta^{*}+\delta \right)-L\left(\vartheta^{*}\right)\right\}\\ \left(c+1\right)\tau {∥\delta_{E_{+}}∥}_{1}+\left(1-\tau \right)\sum_{j\in G_{+}}{∥\delta_{j}∥}_{2}\geq \left(c-1\right)\tau {∥\delta_{E^{c}}∥}_{1}+\left(1-\tau \right)\sum_{j\notin G}{∥\delta_{j}∥}_{2}\\ \;\frac{\tau }{1-\tau }{∥\delta_{E_{+}}∥}_{1}+\sum_{j\in G_{+}}{∥\delta_{j}∥}_{2}\geq C_{0}\left(\frac{\tau }{1-\tau }{∥\delta_{E^{c}}∥}_{1}+\sum_{j\notin G}{∥\delta_{j}∥}_{2}\right). \end{array}$$

□

**Lemma** **A7.**
*Assume that conditions (A1)–(A3) are satisfied. Then*

$$\begin{array}{c} \underset{v\in V}{\sup}\frac{\left|\Delta L(u,v)-E\{\Delta L(u,v)\}\right|}{{∥v∥}_{2}}>\Lambda_{3} \end{array}$$

*with probability at most $2{\left(Kp\right)}^{2\left(s_{e}+K\right)\left(1-c_{3}^{2}\right)}$, where*

$$\begin{array}{c} \Lambda_{3}=\left(1+\sqrt{2}c_{3}\right)\varsigma_{2}\sqrt{\frac{2\left(s_{e}+K\right)\log\left(pK\right)}{N}} \end{array}$$

*and ΔL(u,v)=L(u+v)−L(u) for any $u,v\in R^{K(p+1)}$ and for some constant $c_{3}>1$.*

**Proof.** Given any $u\in R^{K(p+1)}$ and v∈V with $V=\left\{v\in R^{K(p+1)}{|0<∥v∥}_{0}\leq s_{e}+K\right\}$,

$$\begin{array}{cc} \Delta L(u,v)= & \frac{1}{N}\sum_{i=1}^{N}E_{i}^{⊤}\left(\phi_{q}\left\{{\left(U+V\right)}^{⊤}Z_{i}\right\}-\phi_{q}\left\{U^{⊤}Z_{i}\right\}\right)\\ = & \frac{1}{N}\sum_{i=1}^{N}\sum_{k=1}^{K}e_{ik}\left(\phi_{q}\left\{Z_{i}^{⊤}\left(u_{k}+v_{k}\right)\right\}-\phi_{q}\left\{Z_{i}^{⊤}\left(u_{k}\right)\right\}\right)\\ = & \frac{1}{N}\sum_{i=1}^{N}d_{i}\left(u,v\right), \end{array}$$

where u=vec{U}, v=vec{V}. The bounded gradient implies the Lipschitz continuity of $\phi_{q}$ so that $|\phi_{q}\left(u+v\right)-\phi_{q}\left(u\right)|\leq |v|$. Since $e_{ik}\in \left\{0,1\right\}$, we have that

$$\begin{array}{cc} |d_{i}\left(u,v\right)|\leq & \sum_{k=1}^{K}|e_{ik}\left\{\phi_{q}\left\{Z_{i}^{⊤}\left(u_{k}+v_{k}\right)\right\}-\phi_{q}\left(Z_{i}^{⊤}u_{k}\right)\right\}|\\ \leq & \sum_{k=1}^{K}|e_{ik}Z_{i}^{⊤}v_{k}|\leq E_{i}^{⊤}\mathrm{vec}\left\{V^{⊤}Z_{i}\right\}\\ = & v^{⊤}\left(Z_{i}⊗I_{K}\right)E_{i}. \end{array}$$

Note that

$$\begin{array}{c} \sum_{i=1}^{N}{\left(v^{⊤}\left(Z_{i}⊗I_{K}\right)E_{i}\right)}^{2}={∥\mathrm{diag}\left\{\mathrm{vec}\left\{E^{⊤}\right\}\right\}\left(Z⊗I_{K}\right)v∥}_{2}^{2}. \end{array}$$

By Hoeffding’s inequality, we have that

$$\begin{array}{cc} \; & \mathrm{Pr}\left\{|\frac{1}{N}\sum_{i=1}^{N}d_{i}\left(u,v\right)-E\left(\frac{1}{N}\sum_{i=1}^{N}d_{i}\left(u,v\right)\right)|>t\right\}\\ \leq & 2\exp\left\{-\frac{2N^{2}t^{2}}{4∥\mathrm{diag}\left\{\mathrm{vec}\left\{E^{⊤}\right\}\right\}\left(Z⊗I_{K}\right){v∥}_{2}^{2}}\right\}\\ \leq & 2\exp\left\{-\frac{Nt^{2}}{2\varsigma_{2}^{2}{∥v∥}_{2}^{2}}\right\}. \end{array}$$

Thus $\mathrm{Pr}\left\{R\left(v\right)>\Lambda_{3}\right\}\leq 2{\left(Kp\right)}^{-\left(s_{e}+K\right)c_{3}^{2}}$ with

$$\begin{array}{c} R\left(v\right)=\frac{\left|\Delta L(u,v)-E\{\Delta L(u,v)\}\right|}{{∥v∥}_{2}}\;\mathrm{and}\;\Lambda_{3}=c_{3}\varsigma_{2}\sqrt{\frac{2\left(s_{e}+K\right)\log\left(pK\right)}{N}}. \end{array}$$

Next, we consider covering V with ϵ-balls such that for any $v_{1}$ and $v_{2}$ in the same ball, $|\frac{v_{1}}{∥v_{1}{∥}_{2}}-\frac{v_{1}}{∥v_{1}{∥}_{2}}|\leq \epsilon ,$ where ϵ is a small positive number. The number of ϵ-balls required to cover a *m*-dimensional unit ball is bounded by ${\left(\frac{2}{\epsilon }+1\right)}^{m}$. Then for those $\frac{v}{{∥v∥}_{2}}$, we require a covering number of at most ${\left(3\left(Kp\right)/\epsilon \right)}^{s_{e}+K}$. Let N denote such an ϵ-net. We have that

$$\begin{array}{c} \mathrm{Pr}\left\{\underset{v\in N}{\sup}R\left(v\right)>\Lambda_{3}\right\}\leq {\left(\frac{3Kp}{\epsilon }\right)}^{s_{e}+K}2{\left(Kp\right)}^{-\left(s_{e}+K\right)c_{3}^{2}}=2{\left\{\frac{3}{\epsilon }{\left(Kp\right)}^{1-c_{3}^{2}}\right\}}^{s_{e}+K}. \end{array}$$

Furthermore, for any $v_{1},v_{2}\in V$,

$$\begin{array}{cc} |R\left(v_{1}\right)-R\left(v_{2}\right)|\leq & \frac{2}{N}∥\mathrm{diag}\left\{\mathrm{vec}\left\{E^{⊤}\right\}\right\}\left(Z⊗I_{K}\right)\left(\frac{v_{1}}{∥v_{1}{∥}_{2}}-\frac{v_{1}}{∥v_{1}{∥}_{2}}\right){∥}_{1}\\ \leq & \frac{2}{\sqrt{N}}∥\mathrm{diag}\left\{\mathrm{vec}\left\{E^{⊤}\right\}\right\}\left(Z⊗I_{K}\right)\left(\frac{v_{1}}{∥v_{1}{∥}_{2}}-\frac{v_{1}}{∥v_{1}{∥}_{2}}\right){∥}_{2}\\ \leq & 2\varsigma_{2}\epsilon . \end{array}$$

Therefore $\sup_{v\in V}R\left(v\right)\leq \sup_{v\in N}R\left(v\right)+2\varsigma_{2}\epsilon$. Taking $\epsilon =\sqrt{\frac{\left(s_{e}+K\right)\log\left(pK\right)}{2N}}$, we have that

$$\begin{array}{cc} \mathrm{Pr}\left\{\underset{v\in V}{\sup}R\left(v\right)>\Lambda_{3}\right\}\leq & \mathrm{Pr}\left\{\underset{v\in N}{\sup}R\left(v\right)>\left(c_{3}-1\right)\varsigma_{1}\sqrt{\frac{2\left(s_{e}+K\right)\log\left(pK\right)}{N}}\right\}\\ \leq & 2{\left\{\sqrt{\frac{2N}{\left(s_{e}+K\right)\log\left(pK\right)}}3{\left(Kp\right)}^{1-{\left(c_{3}-1\right)}^{2}}\right\}}^{s_{e}+K}\\ \leq & 2{\left\{{\left(Kp\right)}^{2-{\left(c_{3}-1\right)}^{2}}\right\}}^{s_{e}+K}. \end{array}$$

Setting $c_{3}=1+\sqrt{2}c_{4}$ and $c_{4}>1$, we obtain the desired result that

$$\begin{array}{c} \mathrm{Pr}\left\{\underset{v\in V}{\sup}R\left(v\right)>\left(1+\sqrt{2}c_{4}\right)\varsigma_{2}\sqrt{\frac{2\left(s_{e}+K\right)\log\left(pK\right)}{N}}\right\}\leq 2{\left(Kp\right)}^{2\left(s_{e}+K\right)\left(1-c_{4}^{2}\right)}. \end{array}$$

□

**Proof** **of** **Theorem** **2.** Consider a disjoint partition on the coordinate set $\delta =\hat{\vartheta }-\vartheta^{*}$, that is, $\delta =\sum_{m=1}^{M}v_{m}$ with $v_{m}\in V$. Note that, each subvector $v_{m}$ has at most $s_{e}+K$ non-zero coordinates. Denote $v_{0}=0$ and $u_{m}=\vartheta^{*}+\sum_{l=0}^{m-1}v_{l}$ so that $u_{1}=\vartheta^{*}$ and $u_{M}+v_{M}=\vartheta^{*}+\delta$. We have the decomposition

$$\begin{array}{cc} \Delta L(\vartheta^{*},\delta )= & L\left(\vartheta^{*}+\sum_{m=1}^{M}v_{m}\right)-L\left(\vartheta^{*}\right)=\sum_{m=1}^{M}L\left(\vartheta^{*}+\sum_{l=0}^{m}v_{l}\right)-L\left(\vartheta^{*}+\sum_{l=0}^{m-1}v_{l}\right)\\ = & \sum_{m=1}^{M}L\left(u_{m}+v_{m}\right)-L\left(u_{m}\right)=\sum_{m=1}^{M}\Delta L\left(u_{m},v_{m}\right). \end{array}$$

By Lemma A7,

$$\begin{array}{c} \sum_{m=1}^{M}\Delta L\left(u_{m},v_{m}\right)\geq \sum_{m=1}^{M}E\left\{\Delta L(u_{m},v_{m})\right\}-\Lambda_{3}∥v_{m}{∥}_{2}=E\left\{\Delta L(\vartheta^{*},\delta )\right\}-\Lambda_{3}{∥\delta ∥}_{2} \end{array}$$

with high probability. By Lemma A5, L is twice differentiable so that

$$\begin{array}{cc} E\left\{\Delta L(\vartheta^{*},\delta )\right\} & =\frac{1}{N}\sum_{i=1}^{N}E\left(E_{i}^{⊤}\phi_{q}\left\{F_{i}\left(\vartheta^{*}+\delta \right)\right\}\right)-E\left(E_{i}^{⊤}\phi_{q}\left\{F_{i}\left(\vartheta^{*}\right)\right\}\right)\\ & =L\left(\vartheta^{*}+\delta \right)-L\left(\vartheta^{*}\right)\\ & =S{\left(\vartheta^{*}\right)}^{⊤}\delta +\frac{1}{2}\delta^{⊤}H\left(\vartheta^{*}\right)\delta +o\left({∥\delta ∥}_{2}^{2}\right)\\ & \geq 0+\frac{\varsigma_{3}^{2}}{2}{∥\delta ∥}_{2}^{2}+o\left({∥\delta ∥}_{2}^{2}\right). \end{array}$$

Consequently, $\Delta L(\vartheta^{*},\delta )$ is bounded below by $\frac{\varsigma_{3}^{2}}{2}{∥\delta ∥}_{2}^{2}-\Lambda_{3}{∥\delta ∥}_{2}$ with high probability. Note that

$$\begin{array}{cc} P\left(\beta^{*}\right)-P\left(\beta^{*}+\tilde{\delta }\right)\leq & \tau \left({∥\delta_{E_{+}}∥}_{1}-{∥\delta_{E^{c}}∥}_{1}\right)+\left(1-\tau \right)\left(\sum_{j\in G_{+}}{∥\delta_{j}∥}_{2}-\sum_{j\notin G}{∥\delta_{j}∥}_{2}\right)\\ \leq & \left(\tau {∥\delta_{E_{+}}∥}_{1}+\left(1-\tau \right)\sum_{j\in G_{+}}{∥\delta_{j}∥}_{2}\right). \end{array}$$

From (A8),

$$\begin{array}{cc} L\left(\vartheta^{*}\right)+\lambda P\left(\beta^{*}\right)\geq & L\left(\hat{\vartheta }\right)+\lambda P\left(\hat{\beta }\right)\\ \lambda \left\{P\left(\beta^{*}\right)-P\left(\beta^{*}+\tilde{\delta }\right)\right\}\geq & L\left(\vartheta^{*}+\delta \right)-L\left(\vartheta^{*}\right)\\ \lambda \left(\tau {∥\delta_{E_{+}}∥}_{1}+\left(1-\tau \right)\sum_{j\in G_{+}}{∥\delta_{j}∥}_{2}\right)\geq & \frac{\varsigma_{3}^{2}}{2}{∥\delta ∥}_{2}^{2}-\Lambda_{3}{∥\delta ∥}_{2}. \end{array}$$

Clearly, ${∥\delta_{E_{+}}∥}_{1}\leq \sqrt{s_{e}+K}{∥\delta_{E_{+}}∥}_{2}\leq \sqrt{s_{e}+K}{∥\delta ∥}_{2}$ and $\sum_{j\in G_{+}}{∥\delta_{j}∥}_{2}\leq \sqrt{s_{g}+1}{∥\delta ∥}_{2}$. We conclude that

$$\begin{array}{cc} \frac{\varsigma_{3}^{2}}{2}{∥\delta ∥}_{2}^{2}\leq & \lambda \left(\tau {∥\delta_{E_{+}}∥}_{1}+\left(1-\tau \right)\sum_{j\in G_{+}}{∥\delta_{j}∥}_{2}\right)+\Lambda_{3}{∥\delta ∥}_{2}\\ {∥\delta ∥}_{2}^{2}\leq & 2\varsigma_{3}^{-2}\left\{\lambda \left(\tau \sqrt{s_{e}+K}+\left(1-\tau \right)\sqrt{s_{g}+1}\right)+\Lambda_{3}\right\}{∥\delta ∥}_{2}, \end{array}$$

after which the desired result follows from straightforward algebraic manipulation. □

### Appendix A.5. Proof of Lemma 3

**Proof.** Since

$$\begin{array}{c} \mathrm{vec}{\left(F^{⊤}\right)}^{⊤}=\mathrm{vec}{\left\{{\left(1_{N}\alpha^{⊤}+\mathrm{XB}\right)}^{⊤}\right\}}^{⊤}=\alpha^{⊤}\left(1_{N}^{⊤}⊗I_{K}\right)+\mathrm{vec}{\left(B^{⊤}\right)}^{⊤}\left(X^{⊤}⊗I_{K}\right), \end{array}$$

we have that

$$\begin{array}{c} \left\{\begin{array}{c} \frac{\partial \mathrm{vec}{\left(F^{⊤}\right)}^{⊤}}{\partial \alpha }=\frac{\partial \alpha^{⊤}\left(1_{N}^{⊤}⊗I_{K}\right)}{\partial \alpha }=\frac{\partial \alpha^{⊤}}{\partial \alpha }\left(1_{N}^{⊤}⊗I_{K}\right)=I_{K}\left(1_{N}^{⊤}⊗I_{K}\right)=1_{N}^{⊤}⊗I_{K}\\ \frac{\partial \mathrm{vec}{\left(F^{⊤}\right)}^{⊤}}{\partial \mathrm{vec}(B^{⊤})}=\frac{\partial \mathrm{vec}{\left(B^{⊤}\right)}^{⊤}\left(X^{⊤}⊗I_{K}\right)}{\partial \mathrm{vec}(B^{⊤})}=I_{pK}\left(X^{⊤}⊗I_{K}\right)=X^{⊤}⊗I_{K}. \end{array}\right. \end{array}$$

The derivative with respect to α is

$$\begin{array}{cc} NS(\alpha )= & N\frac{\partial L(\theta )}{\partial \alpha }=\frac{\partial }{\partial \alpha }\mathrm{vec}{\left\{E^{⊤}\right\}}^{⊤}\mathrm{vec}\left\{\phi_{q}\left(F^{⊤}\right)\right\}\\ = & \frac{\partial \mathrm{vec}{\left(F^{⊤}\right)}^{⊤}}{\partial \alpha }\frac{\partial \phi_{q}\left\{\mathrm{vec}{\left(F^{⊤}\right)}^{⊤}\right\}}{\partial \mathrm{vec}(F^{⊤})}\mathrm{vec}\left\{E^{⊤}\right\}\\ = & \left(1_{N}^{⊤}⊗I_{K}\right)\mathrm{diag}\left(\mathrm{vec}\left\{\phi_{q}^{\prime }\left(F^{⊤}\right)\right\}\right)\mathrm{vec}\left\{E^{⊤}\right\}\\ = & \mathrm{vec}\left(I_{K}{\left\{E\circ \phi_{q}^{\prime }\left(F\right)\right\}}^{⊤}1_{N}\right)\\ = & {\left\{E\circ \phi_{q}^{\prime }\left(F\right)\right\}}^{⊤}1_{N}. \end{array}$$

Thus,

$$\begin{array}{cc} {∥S\left(\alpha \right)|}_{v}^{u}{∥}_{2}^{2}= & ∥S\left(u\right)-S\left(v\right){∥}_{2}^{2}=N^{-2}∥\left(1_{N}^{⊤}⊗I_{K}\right)\mathrm{vec}\left\{{\left(E\circ \phi_{q}^{\prime }\left\{F\left(\alpha \right)\right\}{|}_{v}^{u}\right)}^{⊤}\right\}{∥}_{2}^{2}\\ \leq & N^{-2}∥1_{N}^{⊤}⊗I_{K}{∥}_{2}^{2}∥\mathrm{vec}\left\{{\left(E\circ \phi_{q}^{\prime }\left\{F\left(\alpha \right)\right\}{|}_{v}^{u}\right)}^{⊤}\right\}{∥}_{2}^{2}\\ = & N^{-1}\sum_{k=1}^{K}\sum_{i=1}^{N}e_{ik}^{2}{\left(\phi_{q}^{\prime }\left\{f_{ik}\left(u_{k}\right)\right\}-\phi_{q}^{\prime }\left\{f_{ik}\left(v_{k}\right)\right\}\right)}^{2}\\ \leq & N^{-1}\sum_{k=1}^{K}\left(\sum_{i=1}^{N}e_{ik}\right)L_{q}^{2}{\left(u_{k}-v_{k}\right)}^{2}\\ \leq & N^{-1}n_{\max}L_{q}^{2}{∥u-v∥}_{2}^{2}, \end{array}$$

where $L_{q}=\frac{{\left(q+1\right)}^{2}}{q}$ is the Lipschitz constant of $\phi_{q}^{\prime }$. We have that $L_{\alpha }=\sqrt{\frac{n_{\max}}{N}}L_{q}$. The derivative with respect to $\mathrm{vec}(B^{⊤})$ is

$$\begin{array}{cc} N\frac{\partial L(\theta )}{\partial \mathrm{vec}(B^{⊤})}= & \frac{\partial }{\partial \mathrm{vec}(B^{⊤})}\mathrm{vec}{\left\{E^{⊤}\right\}}^{⊤}\mathrm{vec}\left\{\phi_{q}\left(F^{⊤}\right)\right\}\\ = & \frac{\partial \mathrm{vec}{\left(F^{⊤}\right)}^{⊤}}{\partial \mathrm{vec}(B^{⊤})}\frac{\partial \phi_{q}\left\{\mathrm{vec}{\left(F^{⊤}\right)}^{⊤}\right\}}{\partial \mathrm{vec}(F^{⊤})}\mathrm{vec}\left\{E^{⊤}\right\}\\ = & \left(X^{⊤}⊗I_{K}\right)\mathrm{diag}\left(\mathrm{vec}\left\{\phi_{q}^{\prime }\left(F^{⊤}\right)\right\}\right)\mathrm{vec}\left\{E^{⊤}\right\}\\ = & \mathrm{vec}\left(I_{K}{\left\{E\circ \phi_{q}^{\prime }\left(F\right)\right\}}^{⊤}X\right)\\ = & \mathrm{vec}\left({\left\{E\circ \phi_{q}^{\prime }\left(F\right)\right\}}^{⊤}X\right). \end{array}$$

Therefore, the derivative with respect to B is $S\left(B\right)=N^{-1}X^{⊤}\left\{E\circ \phi_{q}^{\prime }\left(F\right)\right\}$. Note that

$$\begin{array}{cc} \mathrm{vec}\left(X^{⊤}\left\{E\circ \phi_{q}^{\prime }\left(F\right)\right\}\right)= & \left(I_{K}⊗X^{⊤}\right)\mathrm{diag}\left\{\mathrm{vec}\left(E\right)\right\}\mathrm{vec}\left\{\phi_{q}^{\prime }\left(F\right)\right\}\\ = & \left\{\overset{K}{\underset{k=1}{⨁}}X^{⊤}\mathrm{diag}\left(e_{k}\right)\right\}\mathrm{vec}\left\{\phi_{q}^{\prime }\left(F\right)\right\} \end{array}$$

and

$$\begin{array}{c} \sum_{i=1}^{N}{\left\{e_{ik}X_{i}^{⊤}\left(u_{k}-v_{k}\right)\right\}}^{2}={∥\mathrm{diag}\left(e_{k}\right)X\left(u_{k}-v_{k}\right)∥}_{2}^{2}\leq ∥\mathrm{diag}\left(e_{k}\right){X∥}_{2}^{2}{∥u_{k}-v_{k}∥}_{2}^{2}; \end{array}$$

thus

$$\begin{array}{cc} N^{2}∥\mathrm{vec}\left\{S\left(U\right)-S\left(V\right)\right\}{∥}_{2}^{2}= & \sum_{k=1}^{K}∥X^{⊤}\mathrm{diag}\left(e_{k}\right)\phi_{q}\left\{f_{k}\left(b_{k}\right)\right\}{|}_{v_{k}}^{u_{k}}{∥}_{2}^{2}\\ \leq & \sum_{k=1}^{K}∥X^{⊤}\mathrm{diag}\left(e_{k}\right){∥}_{2}^{2}∥\mathrm{diag}\left(e_{k}\right)\phi_{q}\left\{f_{k}\left(b_{k}\right)\right\}{{|}_{v_{k}}^{u_{k}}∥}_{2}^{2}\\ \leq & \sum_{k=1}^{K}{∥\mathrm{diag}\left(e_{k}\right)X∥}_{2}^{2}\sum_{i=1}^{N}e_{ik}{\left(\phi_{q}\left\{f_{ik}\left(u_{k}\right)\right\}-\phi_{q}\left\{f_{ik}\left(v_{k}\right)\right\}\right)}^{2}\\ \leq & L_{q}^{2}\sum_{k=1}^{K}{∥\mathrm{diag}\left(e_{k}\right)X∥}_{2}^{2}\sum_{i=1}^{N}{\left\{e_{ik}X_{i}^{⊤}\left(u_{k}-v_{k}\right)\right\}}^{2}\\ \leq & L_{q}^{2}\sum_{k=1}^{K}∥\mathrm{diag}\left(e_{k}\right){X∥}_{2}^{4}{∥u_{k}-v_{k}∥}_{2}^{2}\\ \leq & \max_{k}{\left\{∥\mathrm{diag}\left(e_{k}\right){X∥}_{2}^{2}\right\}}^{2}{∥\mathrm{vec}\left(U-V\right)∥}_{2}^{2}. \end{array}$$

We conclude that $L_{B}=L_{q}N^{-1}\max_{k}{∥\mathrm{diag}\left(e_{k}\right)X∥}_{2}^{2}$. □

### Appendix A.6. Proof of Theorem 3

**Lemma** **A8.**
*The indicator function*

$$\begin{array}{c} \delta_{R}\left(x\right)=\left\{\begin{array}{cc} 0, & \mathrm{if}\;\;x\in R\\ \infty , & \mathrm{if}\;\;x\notin R, \end{array}\right. \end{array}$$

*where $R=\{x\in R^{p}|1_{p}^{⊤}x=0\}$, has subdifferential*

$$\begin{array}{c} \partial \delta_{R}\left(x\right)=\left\{\begin{array}{cc} \{g\in R^{p}|g=s1_{p},s\in R\}, & \mathrm{if}\;\;x\in R\\ ⌀, & \mathrm{if}\;\;x\notin R. \end{array}\right. \end{array}$$

**Proof.** Suppose that x∈R. Then $g\in \partial \delta_{R}\left(x\right)$ if and only if both

$$\begin{array}{c} \delta_{R}\left(y\right)\geq \delta_{R}\left(x\right)+\langle g,y-x\rangle \;\mathrm{for}\;\mathrm{all}\;y\in R\;\mathrm{and}\\ \omega^{⊤}\left(y-x\right)\leq 0. \end{array}$$

Let z=y−x. Then z∈R since $1_{p}^{⊤}\left(y-X\right)=0$. Thus, $g^{⊤}z\leq 0$. If $g^{⊤}z=0$, then $g\in \{g\in R^{p}|g=s1_{p},\;s\in R\}$. If there exists $g\in \partial \delta_{R}\left(x\right)$ satisfying $g^{⊤}z<0$ for some z∈R, then −z∈R, so we must have that $g^{⊤}z>0$. This is a contradiction. Now, for any x∉R, we have that $g\in \partial \delta_{R}\left(x\right)$ if and only if both

$$\begin{array}{c} \delta_{R}\left(y\right)\geq \delta_{R}\left(x\right)+\langle g,y-x\rangle \;\mathrm{for}\;\mathrm{all}\;y\in R\;\mathrm{and}\\ \omega^{⊤}\left(x-y\right)\geq \infty . \end{array}$$

For x∉R and y∈R, since $z=x-y\in R^{p}$ and $g^{⊤}z\geq \infty$, it must be that g∈⌀. □

**Proof** **of** **Theorem** **3.** It is sufficient to minimize the objective function

$$\begin{array}{c} G\left(\beta \right)=\frac{1}{2}∥\beta -\beta_{*}{∥}_{2}^{2}+\rho_{1}{∥\beta ∥}_{1}+\rho_{2}{∥\beta ∥}_{2}+\delta_{R}\left(\beta \right), \end{array}$$

where $R=\{x\in R^{K}|1_{K}^{⊤}\beta =0\}$. Then the subdifferential of G(β) is

$$\begin{array}{c} \partial G\left(\beta \right)=\beta -\beta_{*}+\rho_{1}{\partial ∥\beta ∥}_{1}+\rho_{2}\partial {∥\beta ∥}_{2}+\partial \delta_{R}\left(\beta \right). \end{array}$$

For an optimal solution $\beta^{*}\in R$, we have that $0_{p}\in \partial G\left(\beta^{*}\right)$ if and only if there exist $u\in {\partial ∥\beta ∥}_{1}$, $v\in {\partial ∥\beta ∥}_{2}$ and s∈R such that $\beta^{*}=\beta_{*}-\rho_{1}u-\rho_{2}v-s1_{p}$. Since $1^{⊤}\beta^{*}=0$, we have that $s=p^{-1}1_{p}^{⊤}\left(\beta_{*}-\rho_{1}u-\rho_{2}v\right)$, so

$$\begin{array}{cc} \beta^{*} & =P_{K}\left(\beta_{*}-\rho_{1}u-\rho_{2}v\right). \end{array}$$

If $\beta^{*}=0_{p}$, then $|u_{j}|<1$ for j=1,…,p, ${∥v∥}_{2}\leq 1$ and

$$\begin{array}{c} ∥P_{K}\left(\beta_{*}-\rho_{1}u\right){∥}_{2}=\rho_{2}∥P_{K}{v∥}_{2}\leq \rho_{2}∥P_{K}{∥}_{2}{∥v∥}_{2}=\rho_{2}{∥v∥}_{2}\leq \rho_{2}; \end{array}$$

If $\beta^{*}\neq 0_{K}$, then $u\in {\partial ∥X∥}_{1}$, $v=\frac{\beta^{*}}{∥\beta^{*}{∥}_{2}}$ and

$$\begin{array}{cc} \beta^{*}= & \;P_{K}\left(\beta_{*}-\rho_{1}u-\rho_{2}\frac{\beta^{*}}{∥\beta^{*}{∥}_{2}}\right)\\ \left(1+\frac{\rho_{2}}{∥\beta^{*}{∥}_{2}}\right)\beta^{*}= & \;P_{K}\left(\beta_{*}-\rho_{1}u\right). \end{array}$$

Note that $\beta^{*}=P_{K}\beta^{*}\in R$. Taking the norm of both sides, we see that

$$\begin{array}{cc} \left(1+\frac{\rho_{2}}{∥\beta^{*}{∥}_{2}}\right){∥\beta^{*}∥}_{2} & =∥P_{K}\left(\beta_{*}-\rho_{1}u\right){∥}_{2}\\ ∥\beta^{*}{∥}_{2} & =∥P_{K}\left(\beta_{*}-\rho_{1}u\right){∥}_{2}-\rho_{2}>0. \end{array}$$

Substituting this result back into the $\beta^{*}\neq 0_{K}$ case, we have that

$$\begin{array}{c} \beta^{*}=\left\{1-\frac{\rho_{2}}{∥P_{K}\left(\beta_{*}-\rho_{1}u\right){∥}_{2}}\right\}P_{K}\left(\beta_{*}-\rho_{1}u\right). \end{array}$$

Combining the above two cases gives the desired result. □

### Appendix A.7. Proof of Theorem 4

**Proof.** Denote the objective function by

$$\begin{array}{c} G\left(b\right)=\frac{1}{2}{\left(b-t\right)}^{2}+\varrho \left\{|b|+|b+s|\right\}. \end{array}$$

When s=0, we obtain a lasso problem with

$$\begin{array}{c} b^{*}=\underset{b\in R}{\mathrm{argmin}}\frac{1}{2}{\left(b-t\right)}^{2}+2\varrho \left|x\right|=S\left(t,2\varrho \right). \end{array}$$

When s≠0, the subdifferential of G(b) is

$$\begin{array}{c} \partial G(b)=b-t+\varrho \{\partial |x|+\partial |x+s|\}. \end{array}$$

We see that $0\in \partial G(b^{*})$ if and only if there exist u∈∂|b| and v∈∂|b+s| with

$$\begin{array}{c} b^{*}=b-\varrho \left(u+v\right). \end{array}$$

If $b^{*}=0$, then |u|<1 and v=sign(s), hence

$$\begin{array}{c} b^{*}=0\;\mathrm{if}\;\left|t-\varrho \mathrm{sign}\left(s\right)\right|\leq \varrho . \end{array}$$

If s>0, then sign(s)=1 and 0≤t≤2ϱ. If s<0, then sign(s)=−1, and −2ϱ≤t≤0. Note that if t≠0, then sign(s)=sign(t) or sign(s)sign(t)=1. When $b^{*}=-s$, then u=−sign(s) and |v|<1, hence

$$\begin{array}{c} b^{*}=-s\;\mathrm{if}\;\left|t+s+\varrho \mathrm{sign}\left(s\right)\right|\leq \varrho . \end{array}$$

If s>0, then sign(s)=1 and −(s+2λ)≤t≤−s<0. If s<0, then sign(s)=−1 and 0<−s≤t≤−(s−2λ). Note that sign(s)=−sign(t) is equivalent to sign(s)sign(t)=−1. Let $C\left(s,t\right)=\frac{1-\mathrm{sign}(s)\mathrm{sign}(t)}{2}\left|s\right|\geq 0$. We can summarize the two cases above as

(A9) $$\begin{array}{c} b^{*}=-C\left(s,t\right)\;\mathrm{if}\;0\leq C\left(s,t\right)\leq |t|\leq C(s,t)+2\varrho . \end{array}$$

If $b^{*}\neq 0,-s$, then $u=\mathrm{sign}(b^{*})$ and $v=\mathrm{sign}(b^{*}+s)$, thus

$$\begin{array}{c} b^{*}=t-\varrho \left\{\mathrm{sign}\left(b^{*}\right)+\mathrm{sign}\left(b^{*}+s\right)\right\}\\ b^{*}+s=t+s-\varrho \left\{\mathrm{sign}\left(b^{*}\right)+\mathrm{sign}\left(b^{*}+s\right)\right\}. \end{array}$$

If $\mathrm{sign}\left(b^{*}\right)=-\mathrm{sign}\left(b^{*}+s\right)=1$, then $b^{*}\left(b^{*}+s\right)<0$ or 0<t<−s. Thus $b^{*}=t>0$ if 0<t<−s. If $\mathrm{sign}\left(b^{*}\right)=-\mathrm{sign}\left(b^{*}+s\right)=-1$, then $b^{*}\left(b^{*}+s\right)<0$ or −s<t<0. Thus $b^{*}=t<0$ if −s<t<0. Rewriting the two cases above, we have that

(A10) $$\begin{array}{c} b^{*}=t\;\;\mathrm{if}\;0<\left|t\right|<C\left(s,t\right). \end{array}$$

If $\mathrm{sign}\left(b^{*}\right)=\mathrm{sign}\left(b^{*}+s\right)=1$, then

$$\begin{array}{c} \min\{b^{*},b^{*}+s\}>0\\ t-2\varrho +\frac{s-|s|}{2}>0\\ \mathrm{sign}\left(t\right)|t|>\mathrm{sign}\left(t\right)\left(\frac{\left|s\right|}{2}+2\varrho \right)-\frac{s}{2}>0. \end{array}$$

Note that t>0 and sign(x)=sign(t). If $\mathrm{sign}\left(b^{*}\right)=\mathrm{sign}\left(b^{*}+s\right)=-1$, then

$$\begin{array}{c} \max\{b^{*},b^{*}+s\}<0\\ t+2\varrho +\frac{s+|s|}{2}>0\\ \mathrm{sign}\left(t\right)|t|<\mathrm{sign}\left(t\right)\left(\frac{\left|s\right|}{2}+2\varrho \right)-\frac{s}{2}<0. \end{array}$$

Note that t<0 and sign(x)=sign(t). Rewriting the two cases above, we have that

(A11) $$\begin{array}{c} b^{*}=t-2\varrho \mathrm{sign}\left(t\right)\;\mathrm{if}\;\left|t\right|>2\varrho +C\left(s,t\right). \end{array}$$

Summarizing (A9)–(A11),

$$\begin{array}{c} b^{*}=\left\{\begin{array}{cc} t, & |t|<C(s,t),\\ -C(s,t), & C(s,t)\leq |t|\leq C(s,t)+2\varrho ,\\ \mathrm{sign}(t)(|t|-2\varrho ), & |t|>C(s,t)+2\varrho , \end{array}\right. \end{array}$$

with $C\left(s,t\right)=\frac{1-\mathrm{sign}(s)\mathrm{sign}(t)}{2}\left|s\right|\geq 0$. On one hand, when s≠0,

$$\begin{array}{c} b^{*}=t-S\left(t,C(s,t)\right)+S\left\{S\left(t,C(s,t)\right),2\varrho \right\}. \end{array}$$

On the other hand, when s=0, it follows that $b^{*}=S\left(t,2\varrho \right)$ since S(z,0)=z. □
